# ASPM, CDC20, DLGAP5, BUB1B, CDCA8, and NCAPG May Serve as Diagnostic and Prognostic Biomarkers in Endometrial Carcinoma

**DOI:** 10.1155/2022/3217248

**Published:** 2022-09-17

**Authors:** Qiaoling Zhang, Yingmei Wang, Fengxia Xue

**Affiliations:** ^1^Department of Gynecology and Obstetrics, Tianjin Medical University General Hospital, Tianjin 300000, China; ^2^Tianjin Key Laboratory of Female Reproductive Health and Eugenics, Tianjin Medical University General Hospital, Tianjin 300000, China

## Abstract

Uterine Corpus Endometrial Carcinoma (UCEC), the most common gynecologic malignancy in developed countries, remains to be a major public health problem. Further studies are surely needed to elucidate the tumorigenesis of UCEC. Herein, intersecting 203 differentially expressed genes (DEGs) were identified with the GSE17025, GSE63678, and The Cancer Genome Atlas-UCEC datasets. The Gene Ontology/Kyoto Encyclopedia of Genes and Genomes functional enrichment analysis and protein-protein interaction (PPI) network were performed on those 203 DEGs. Intriguingly, 6 of the top 10 nodes in the PPI network were related to unfavorable prognosis, that is, ASPM, CDC20, DLGAP5, BUB1B, CDCA8, and NCAPG. The mRNA and protein expression levels of the 6 hub genes were elevated in UCEC tissues compared to normal tissues. Higher expression of the 6 hub genes was associated with poor prognostic clinicopathological characteristics. The receiver operating characteristic curve suggested the significant diagnostic ability of the 6 hub genes for UCEC. Then, underlying pathogeneses of UCEC including promoter methylation level, TP53 mutation status, genomic genetic variation, and immune cells infiltration were analyzed. The mRNA expression level of the 6 hub genes was also higher in cervical squamous cell carcinoma and endocervical adenocarcinoma, uterine carcinosarcoma, and ovarian serous cystadenocarcinoma tissues than in corresponding normal tissues. In conclusion, ASPM, CDC20, DLGAP5, BUB1B, CDCA8, and NCAPG may be considered diagnostic and prognostic biomarkers in UCEC.

## 1. Introduction

Uterine Corpus Endometrial Carcinoma (UCEC) is reported as the most common gynecologic malignancy in developed countries [1] and has significant negative impacts on women's physical and mental health.

In 2013, The Cancer Genome Atlas (TCGA) classified UCEC into four molecular subtypes: POLE-ultramutant, microsatellite instability, low copy number variation, and high copy number variation [2]. The risk stratification of UCEC based on the molecular subtypes was the prerequisite for prognostic evaluation. However, it was far from optimizing the treatment guidelines [3]. Further studies are needed to explore suitable biomarkers for purpose of developing more effective treatments and improving the outcome for patients.

This study used bioinformatics techniques to analyze RNA-seq and clinical data of UCEC samples and normal samples obtained from the Gene Expression Omnibus (GEO) and TCGA databases. First, we identified several differentially expressed genes (DEGs) as hub genes. Abnormal spindle microtubule assembly (ASPM) was a centrosomal protein, involved in mitotic spindle formation, neurogenesis, and brain growth [4]. Cell-division cycle protein 20 homolog (CDC20) participated in chromosome segregation and mitotic exit [5]. Discs large-associated protein 5 (DLGAP5), a kinetochore fibers-binding protein, was localized in the mitochondria and might play a vital role in mitophagy [6]. BUB1 mitotic checkpoint serine/threonine kinase B (BUB1B), a component of the spindle assembly checkpoint protein family, interacted with CDC20 to ensure proper chromosome segregation [7]. Human cell division cycle associated 8 (CDCA8) was a member of the chromosome passenger complex (CPC), which was involved in locating the CPC to the centromere, correcting kinetochore binding errors, and stabilizing bipolar spindles [8]. Chromosome-associated Protein G (NCAPG) was crucial for condensing activation by regulating ATPase activity [9]. It had been observed that ASPM, CDC20, DLGAP5, BUB1B, CDCA8, and NCAPG were over-expressed in a diverse array of cancers (including glioblastoma [10–12], lung adenocarcinoma [13–16], prostate cancer [17,18], colorectal cancer [19], breast cancer [20–22], and hepatocellular carcinoma [23–26]), and were linked to adverse prognosis and tumorigenesis. Conversely, a narrow collection of literature was available regarding the role of those genes in UCEC.

Then we verified the differential mRNA and protein expression levels of those hub genes in tumor and normal tissues. Next, we analyzed the hub genes' clinical significance, such as the correlation with clinicopathological features, and diagnostic and prognostic values. Then, underlying pathogeneses of UCEC including promoter methylation level, TP53 mutation status, genomic genetic variation, and immune cells infiltration were analyzed. Finally, we investigated the hub genes mRNA expression in cervical squamous cell carcinoma and endocervical adenocarcinoma (CESC), uterine carcinosarcoma (UCS), and ovarian serous cystadenocarcinoma (OV). We make the case that this study provides good diagnostic and prognostic biomarkers and relevant pathogeneses for UCEC in a new light.

## 2. Materials and Methods

### 2.1. Data Acquisition from GEO, TCGA, and UCSC XENA Data Repository

Two gene expression datasets (GSE17025 and GSE63678) about endometrial cancer were obtained from the GEO database (https://www.ncbi.nlm.nih.gov/geo/) in Dec. 2021. GSE17025 included 91 endometrial cancer samples and 12 normal endometrial samples. The platform of GSE17025 was GPL570((HG-U133_Plus_2) Affymetrix Human Genome U133 Plus 2.0 Array). GSE63678 included 7 endometrial cancer samples and 5 normal endometrial samples. The platform of GSE63678 was GPL571((HG-U133A_2) Affymetrix Human Genome U133A 2.0 Array). The raw RNA expression profile dataset (Workflow Type: HTSeq-Counts/HTSeq-FPKM) about endometrial cancer was downloaded from TCGA (https://portal.gdc.cancer.gov/)-UCEC project in Dec. 2021. RNA-seq data in TPM format about CESC, UCS, OV, and their corresponding normal tissues were obtained from TCGA and GTEx respectively (all downloaded from UCSC Xena (https://xenabrowser.net/datapages/)). 306 CESC samples/13 normal samples, 57 UCS samples/78 normal samples, and 427 OV samples/88 normal samples were extracted in the study.

### 2.2. Data Processing and Identification of DEGs

Pre-processing procedures were used to process raw data in GSE17025 and GSE63678, including the Robust Multichip Average background correction, and completed log2 transformation by “affy” R language package. The probes were converted into the corresponding gene symbol according to the annotation information on the platform. After data pre-processing and standardization, the “limma” R language package was utilized to screen for DEGs between endometrial cancer samples and normal endometrial samples, in which genes with adjusted *P* value<0.05 and |log2 fold change (FC)|>1 were considered as threshold values for identifying DEGs. “Perl” software and “GTF dumps” (downloaded from Ensembl Data (http://grch37.ensembl.org/index.html)) were used for TCGA-UCEC RNA-seq HTSeq-Counts data extraction, integration, and conversion. The “edgeR” R language package was applied to discover DEGs in which genes with adjusted *P* value<0.05 and |log2 FC|>1 were considered the threshold for the DEGs. A Venn diagram tool (http://bioinformatics.psb.ugent.be/webtools/Venn/) was used to identify the co-up-regulated and co-down-regulated DEGs among the above 3 datasets.

### 2.3. Gene Ontology (GO) and Kyoto Encyclopedia of Genes and Genomes (KEGG) Enrichment Analysis of DEGs

All statistical analyses were performed in the R package (V3.6.3). “org.Hs.eg.db” R language package (V3.10.0) was used for ID conversion. The KEGG and GO terms for biological process (BP), cellular component (CC), and molecular function (MF) categories were enriched based on the “clusterProfiler” R language package (V3.14.3) [27] and visualized by the “ggplot2” R language package (V3.3.3).

### 2.4. Protein-Protein Interaction (PPI) Network Construction and Identification of Hub Genes

The Search Tool for the Retrieval of Interacting Genes (STRING) (https://string-db.org) online database was used to predict the PPI network of DEGs. The obtained PPI network was imported into “Cytoscape” software (V3.9.0), and interaction with a combined score ≥0.9 was considered to be statistically significant. CytoHubba plugin tool (Degree method) was applied to identify hub genes.

### 2.5. Data Processing and Validation of the Hub Genes Differential Expression between UCEC Tissues and Normal Tissues

Level 3 HTSeq-FPKM RNA-seq data (including 35 normal tissues and 552 tumor tissues) obtained from the TCGA-UCEC database were transformed into transcripts per million (TPM) reads for further analyses. Scatter plots were generated by the “ggplot2” R language package (V3.3.3) to present differential expression of the hub genes in unpaired samples (35 normal tissues and 552 tumor tissues) and paired samples (23 pairs of tumor/paracancerous tissues) respectively. Besides, the GEPIA (https://gepia.cancer-pku.cn/) database which included 174 tumor tissues and 91 normal tissues (match TCGA normal and GTEx data as normal data) was also applied for validation.

### 2.6. Clinical Significance of the Hub Genes Expression

Kaplan–Meier (KM) survival curve analysis was employed for prognostic analysis including overall survival (OS), and disease-specific survival (DSS). Statistical ranking for the hub genes expression above or below the median value was defined as high or low. Survival data were statistically analyzed by the “Survival” R language package (V3.2-10) and visualized by the “Survminer” R language package (V0.4.9). Unknown follow-up time and outcome were regarded as missing values. The supplementary prognostic data was obtained from a published study [28]. The receiver operating characteristic (ROC) curve was plotted to test the diagnostic performance of the hub genes in UCEC in which the “pROC” R language package (V1.17.0.1) for analysis and the “ggplot2” R language package (V3.3.3) for visualization. Each predictive variable is under its optimal cut-off value.

### 2.7. Protein Expression Analysis of the Hub Genes in the Human Protein Atlas (HPA) and Clinical Proteomic Tumor Analysis Consortium (CPTAC)

HPA (https://www.proteinatlas.org/) website was used to compare the protein expression of the hub genes between normal endometrial tissue and endometrial cancer tissue with the application of the immunohistochemical (IHC) method. Additionally, we conducted UALCAN (https://ualcan.path.uab.edu/analysis.html)-CPTAC to present a throughout analysis of the expression profiles of the hub genes at the protein level.

### 2.8. Correlation Analysis between the Hub Genes Expression and Clinicopathological Features

We performed a stratified analysis based on clinicopathological features (including clinical stage, primary therapy outcome, race, age, weight, height, BMI, histological type, residual tumor, histologic grade, tumor invasion, menopause status, hormones therapy, diabetes, radiation therapy, surgical approach, OS event, DSS event, progress free interval (PFI) event), and determined the relationship between them and the hub genes expression level. “ggplot2” R language package (V3.3.3) for visualization was employed to present the correlation between the hub genes expression and clinical variables (clinical stage and histologic grade). Additionally, single-gene logistics regression analysis was also employed. Statistical ranking for the hub genes expression above or below the median value was defined as high or low, respectively.

### 2.9. The Intrinsic Pathogeneses of UCEC

The UALCAN online tool was applied to present the promoter methylation level of the hub genes based on sample types and expression of the hub genes based on TP53 mutation status. The cBioportal web platform (https://www.cbioportal.org/) was designed for comprehensive genomic analysis. The study “Uterine Corpus Endometrial Carcinoma (TCGA, Nature 2013)” and the genomic profiles (included “Mutations,” “Putative copy number alterations from GISTIC”, and “mRNA expression z-scores relative to diploid samples (RNA Seq V2 RSEM)”) were chosen to analyze. We performed the analysis on mutation spectrum, mutation count, methylation cluster, subtype, and genetic alteration in a total of 232 UCEC samples.

### 2.10. Association between the Hub Genes Expression and Immune Cells Infiltration

The single-sample Gene Set Enrichment Analysis method from the “GSVA” R language package [29] was applied to present the association between different hub genes mRNA expression level and infiltration enrichment of 24 types of immune cells, including dendritic cell (DC), activated DC (aDC), B cells, CD8+ T cells, cytotoxic cells, eosinophils, immature DC (iDC), macrophages, mast cells, neutrophils, NK CD56bright cells, NK CD56dim cells, NK cells, plasmacytoid DC (pDC), T cells, T helper cells, T central memory (Tcm), T effector memory (Tem), T follicular helper (Tfh), T gamma delta (Tgd), Th1 cells, Th17 cells, Th2 cells, and Treg. The immune cells' markers were derived from Immunity [30].

### 2.11. Data Processing and Analysis of the Hub Genes Differential Expression between CESC, UCS, OV Tissues and Corresponding Normal Tissues

RNA-seq data in TPM format were processed uniformly by the Toil process [31] and applied to complete log2 transformation before expression comparison between samples. Differential expression of the hub genes between normal tissues and tumor tissues was visualized by the “ggplot2” R language package (V3.3.3).

### 2.12. Statistical Analysis

All statistical analyses were performed in R (V3.6.3), with *P* values less than 0.05 considered significant. The Wilcoxon rank-sum test and paired-samples *T* test were used to analyze the expression of the hub genes in nonpaired samples and paired samples, respectively. The Cox regression analysis and the KM method were used to evaluate the role of the hub genes expression in UCEC prognosis. Clinicopathological features were compared for high- and low-hub gene expression groups using the Chi-square test, Wilcoxon rank-sum test, *T* test, and Fisher's exact test. The binary logistics model was used for single gene logistics regression analysis. Bonferroni-Dunn test was used to evaluate the relationship between the hub genes expression and clinical stage/histologic grade. Spearman's analysis evaluated the association between the hub genes expression and immune infiltration.

## 3. Results

### 3.1. Identification of DEGs between UCEC Tissues and Normal Tissues

**Figure 1:**
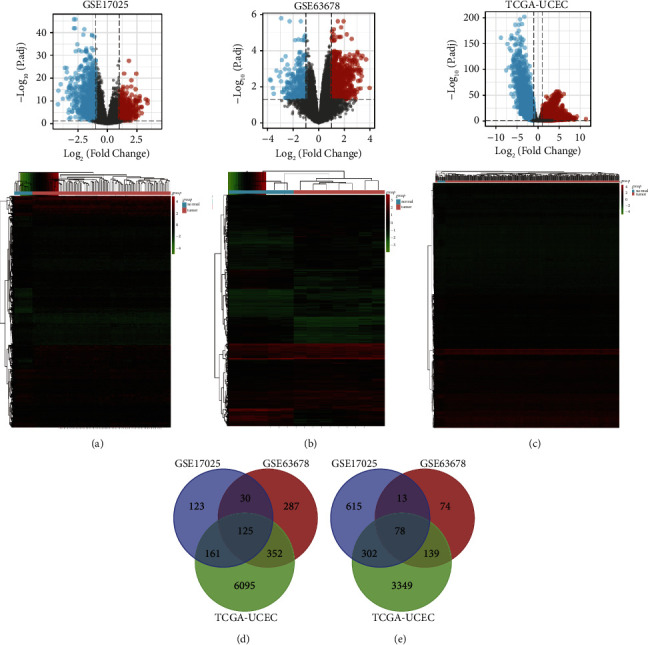
Identification of DEGs (a) Volcano plots and heatmaps of DEGs in the GSE17025 dataset. (b) Volcano plots and heatmaps of DEGs in the GSE63678 dataset. (c) Volcano plots and heatmaps of DEGs in the TCGA-UCEC dataset. (d) co-up-regulated DEGs in the GSE63678, GSE17025, and TCGA-UCEC datasets by the Venn diagram tool. (e) co-down-regulated DEGs in the GSE63678, GSE17025, and TCGA-UCEC datasets by the Venn diagram tool. DEGs, differentially expressed genes.

We identified 1447 DEGs (439 up-regulated and 1008 down-regulated) in the GSE17025 dataset, 1098 DEGs (794 up-regulated and 304 down-regulated) in the GSE63678 dataset, and 10601 DEGs (6733 up-regulated and 3868 down-regulated) in the TCGA-UCEC dataset. Volcano plots and heatmaps were plotted to show the DEGs of the aforementioned 3 datasets respectively (Figures 1(a)–1(c)). With the application of the Venn diagram tool, we identified the intersecting 203 DEGs (125 up-regulated and 78 down-regulated) among the above 3 datasets (Figures 1(d)–1(e)).

### 3.2. GO and KEGG Enrichment Analyses of DEGs

**Figure 2:**
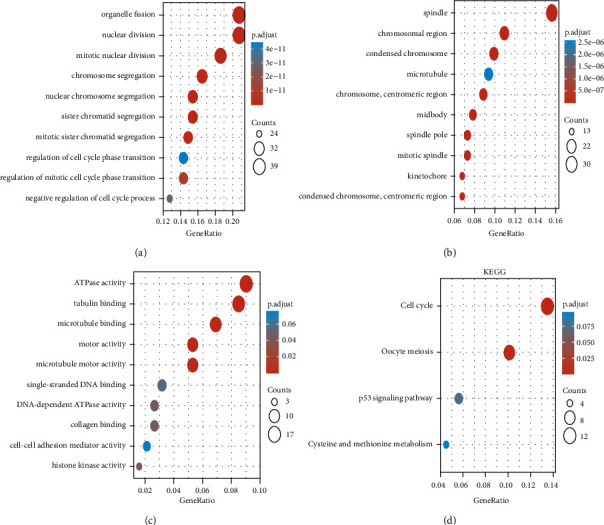
Functional enrichment analysis (a) Enriched GO terms in the BP category. (b) Enriched GO terms in the CC category. (c) Enriched GO terms in the MF category. (d) Enriched KEGG terms. GO, gene ontology; BP, biological process; CC, cellular component; MF, molecular function; KEGG, kyoto encyclopedia of genes and genomes.

A total of 196 Entrez IDs had been successfully converted by “org.Hs.eg.db” R language package (V3.10.0), with a conversion rate of 96.6% (196/203). As shown in Table 1, under the GO functional enrichment analysis, the DEGs were mainly enriched in nuclear division (ontology: BP), the spindle (ontology: CC), and ATPase activity (ontology: MF). Additionally, under the KEGG pathway analysis, the DEGs were enriched in the cell cycle (hsa04110), oocyte meiosis (hsa04114), p53 signaling pathway (hsa04115), and cysteine and methionine metabolism (hsa00270) (Table 2). We vividly presented partial GO/KEGG functional enrichment results in the form of bubble diagrams (Figure 2).

### 3.3. PPI Network and Identification of the Hub Genes

**Figure 3:**
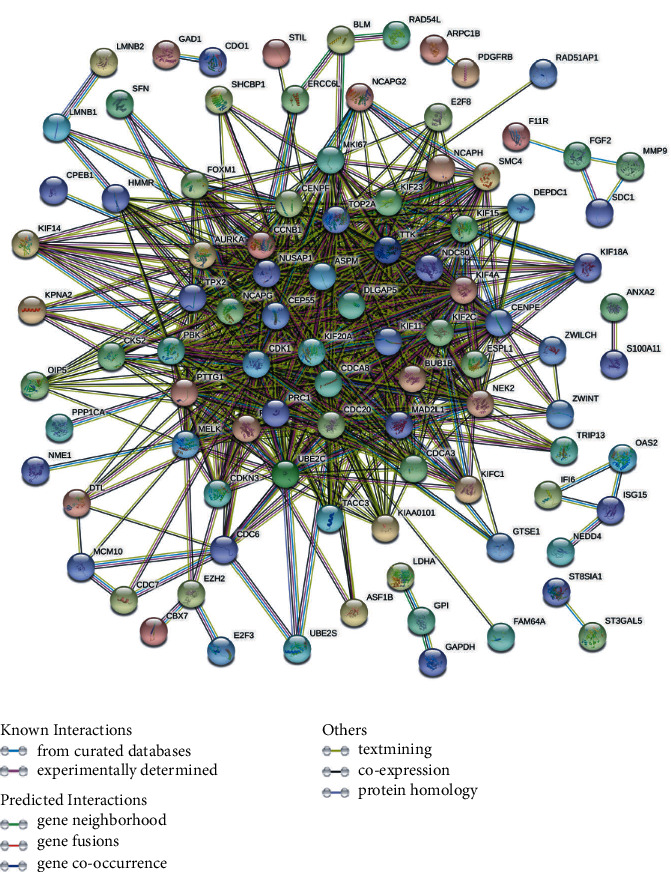
Protein-protein interaction network is constructed by the DEGs. DEGs, differentially expressed genes.

As shown in Figure 3, more than 50 genes interacted closely in the PPI network. The degree of correlation of each node was calculated by the cytoHubba plugin tool. The top 10 nodes ranked by the “Degree” method were located, which included CDK1, ASPM, CCNB1, TOP2A, CDC20, DLGAP5, KIF11, BUB1B, CDCA8, and NCAPG (Table 3).

### 3.4. Abnormal High mRNA Expression of the Hub Genes in UCEC

**Figure 4:**
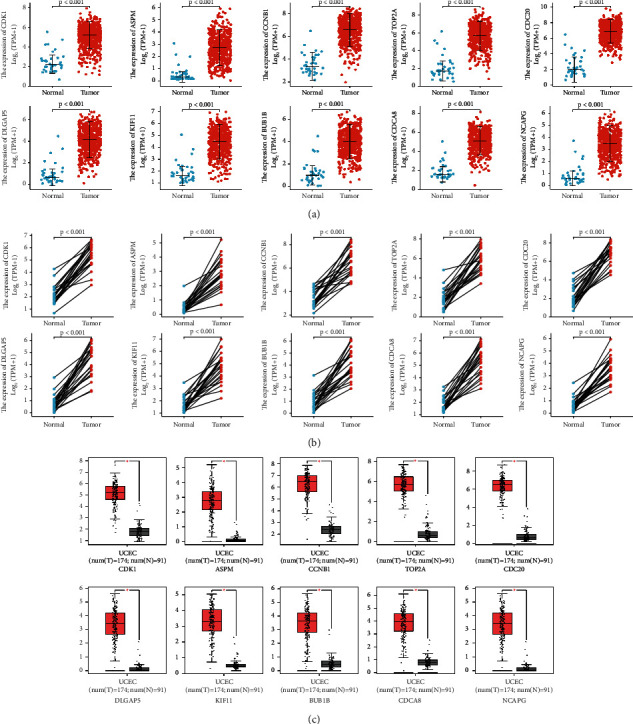
The 10 hub genes mRNA expression level in UCEC (a) The 10 hub genes mRNA expression level in UCEC tissues (*n* = 552) and normal tissues (*n* = 35) in the TCGA-UCEC database. (b) The 10 hub genes mRNA expression level in UCEC tissues (*n* = 23) and matched adjacent normal tissues (*n* = 23) in the TCGA-UCEC database. (c) The 10 hub genes mRNA expression level in UCEC tissues (*n* = 174) and normal tissues (*n* = 91) in the GEPIA database; we use log_2_^(TPM^ ^+^ ^1)^ for log-scale, and red represents UCEC tissues and gray represents normal tissues. UCEC, uterine corpus endometrial carcinoma.

It showed that CDK1, ASPM, CCNB1, TOP2A, CDC20, DLGAP5, KIF11, BUB1B, CDCA8, and NCAPG mRNA expression level in 552 UCEC tissues was dramatically higher than those in 35 normal tissues (*P* < 0.001; Figure 4(a)). In 23 paired samples, 10 hub genes mRNA expression level in UCEC tissues were markedly higher than those in the adjacent normal tissues (*P* < 0.001; Figure 4(b)). Besides, 10 hub genes mRNA expression levels in 174 UCEC tissues were higher than those in 91 normal tissues in the GEPIA database (*P* < 0.05; Figure 4(c)).

### 3.5. 6 Hub Genes over-Expression Was Associated with an Unfavorable Prognosis in UCEC

With the application of KM survival curve analysis, we verified the prediction of the hub genes on clinical outcomes. One sample missed the follow-up time and outcome information was excluded.

**Figure 5:**
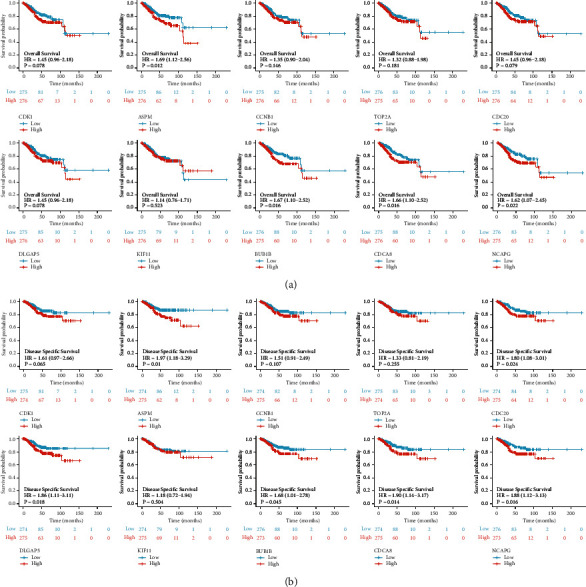
KM survival curve comparing the high and low expression of 10 hub genes in UCEC (a) Overall survival. (b) Disease-specific survival. HR, hazard ratio.

As shown in Figure 5(a), high-ASPM groups (Hazard Ratio (HR) = 1.69, 95% Confidence Interval (CI) = 1.12–2.56, *P*=0.012), high-BUB1B groups (HR = 1.67, 95% CI = 1.10–2.52, *P*=0.016), high-CDCA8 groups (HR = 1.66, 95% CI = 1.10–2.52, *P*=0.016), and high-NCAPG groups (HR = 1.62, 95% CI = 1.07–2.45, *P*=0.022) were all statistically worse than those for the low groups when OS was selected as the prognostic type. With regard to DSS, we found that high-ASPM groups (HR = 1.97, 95% CI = 1.18–3.29, *P*=0.01), high-CDC20 groups (HR = 1.80, 95% CI = 1.08–3.01, *P*=0.024), high-DLGAP5 groups (HR = 1.86, 95% CI = 1.11–3.11, *P*=0.018), high-BUB1B groups (HR = 1.68, 95% CI = 1.01–2.78, *P*=0.045), high-CDCA8 groups (HR = 1.90, 95% CI = 1.14–3.17, *P*=0.014), and high-NCAPG groups (HR = 1.88, 95% CI = 1.12–3.13, *P*=0.016) had a worse DSS (Figure 5(b)).

However, the expression of CDK1, CCNB1, TOP2A, and KIF11 did not affect the OS/DSS of UCEC patients. Overall, these results confirmed that ASPM, CDC20, DLGAP5, BUB1B, CDCA8, and NCAPG obtain prognostic values for UCEC.

### 3.6. Abnormal Protein Expression of the Hub Genes in UCEC

**Figure 6:**
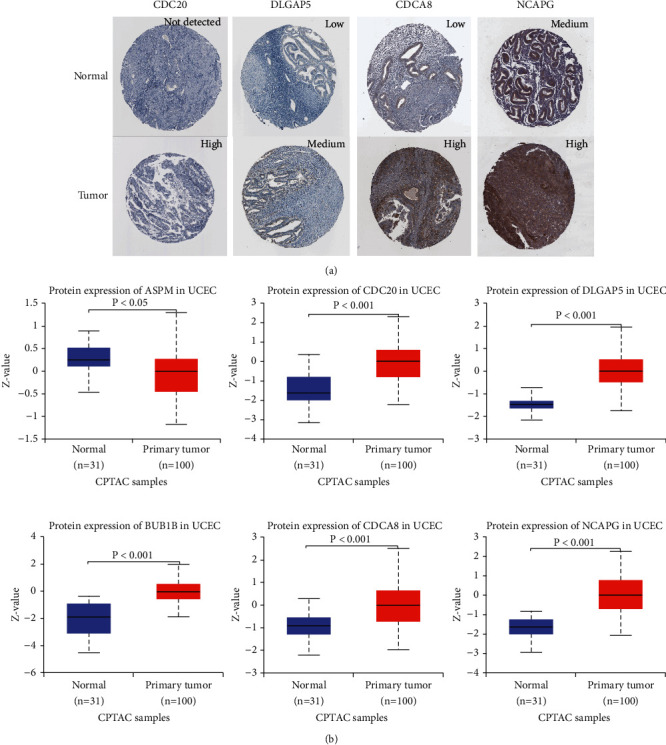
Protein expression of hub genes in UCEC (a) Representative images of 4 hub genes protein expression in UCEC tissues and normal tissues by HPA website. (b) The comparison of 6 hub genes protein expression between UCEC tissues and normal tissues based on the ULCAN-CPTAC platform. UCEC, uterine corpus endometrial carcinoma; HPA, human protein Atlas; CPTAC, clinical proteomic tumor analysis consortium.

We explored the protein expression of the hub genes on the HPA website and representative images were presented in Figure 6(a). By the method of IHC, CDC20 was high staining in UCEC tissues while not detected in normal tissues by antibody CAB004525; DLGAP5 was medium staining in UCEC tissues while low staining in normal tissues by antibody HPA005546; CDCA8 was high staining in UCEC tissues while low staining in normal tissues by antibody HPA028120; NCAPG was high staining in UCEC tissues while medium staining in normal tissues by antibody HPA039613. IHC images of ASPM and BUB1B in UCEC tissues and normal tissues were not found on the HPA website. Furthermore, we used the ULCAN-CPTAC platform to verify the results of protein differential expression obtained from the HPA website. As shown in Figure 6(b), the protein expression level of CDC20 (*P* < 0.001), DLGAP5 (*P* < 0.001), BUB1B (*P* < 0.001), CDCA8 (*P* < 0.001), and NCAPG (*P* < 0.001) was higher in UCEC (*n* = 100) tissues than in normal tissues (*n* = 31), which was consistent with the results obtained from HPA. Interestingly, we discovered that ASPM (*P* < 0.05) exhibited down-regulated pattern at the protein level in UCEC (*n* = 100) tissues compared with normal tissues (*n* = 31). We discussed the contradictory trend in the discussion section in combination with the results of the genetic alteration analysis.

### 3.7. Association between the Hub Genes Expression and Clinicopathological Variables in UCEC

To better understand the role of the hub genes, 552 UCEC samples' clinical information from TCGA was analyzed. The Association between detailed clinicopathologic characteristics of the patients and the 6 hub genes expression was listed in Table S1.

**Figure 7:**
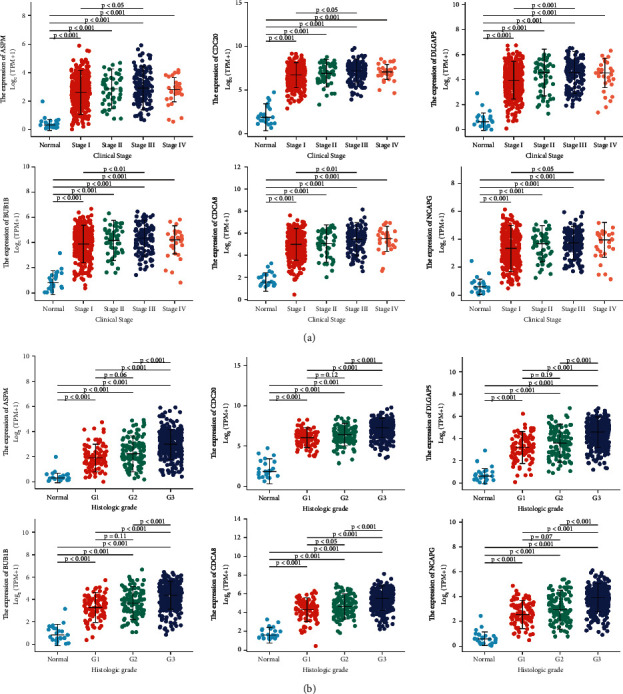
Association of 6 hub genes expression with clinicopathologic characteristics (a) Clinical stage (normal (*n* = 23), stage I (*n* = 342), stage II (*n* = 51), stage III (*n* = 130), stage IV (*n* = 29)). (b) Histologic grade (normal (*n* = 23), G1 (*n* = 98), G2 (*n* = 120), G3 (*n* = 323)). G grade.

Next, we specifically focused on clinical stages (normal (*n* = 23), stage I (*n* = 342), stage II (*n* = 51), stage III (*n* = 130), stage IV (*n* = 29)) and histologic grades (normal (*n* = 23), G1 (*n* = 98), G2 (*n* = 120), G3 (*n* = 323)). As shown in Figure 7(a), patients in more advanced stages tended to express higher mRNA expression of the 6 hub genes (stage III vs. stage I, *P* < 0.05), and the highest mRNA expression of the 6 hub genes was found in stage 3. A lower number of included samples in stage 4 may be a limitation. In addition, it was shown in Figure 7(b) that mRNA expression of the 6 hub genes was significantly related to histologic grades, and the highest mRNA expression of the 6 hub genes was found in grade 3.

Single gene logistics regression analysis illustrated that the hub genes expression as independent variables was associated with poor prognostic clinicopathological characteristics (Table S2). Over-expressed ASPM was positively associated with age (Odds Ratio (OR) = 1.557, 95% CI = 1.101–2.209, *P*=0.013 for >60 vs. ≤ 60), histological type (OR = 2.588, 95% CI = 1.693–4.011, *P* < 0.001 for serous vs. endometrioid), and histologic grade (OR = 3.813, 95% CI = 2.655–5.520, *P* < 0.001 for G3 vs. G1&G2). Over-expressed CDC20 was positively associated with clinical stage (OR = 1.870, 95% CI = 1.288–2.732, *P*=0.001 for stage III & stage IV vs. stage I & stage II), histological type (OR = 4.360, 95% CI = 2.765–7.059, *P* < 0.001 for serous vs. endometrioid), and histologic grade (OR = 5.963, 95% CI = 4.083–8.815, *P* < 0.001 for G3 vs. G1&G2). Over-expressed DLGAP5 was positively associated with clinical stage (OR = 2.339, 95% CI = 1.603–3.441, *P* < 0.001 for stage III & stage IV vs. stage I & stage II), histological type (OR = 3.538, 95% CI = 2.276–5.616, *P* < 0.001 for serous vs. endometrioid), and histologic grade (OR = 5.739, 95% CI = 3.936–8.467, *P* < 0.001 for G3 vs. G1&G2). Over-expressed BUB1B was positively associated with clinical stage (OR = 2.014, 95% CI = 1.385–2.947, *P* < 0.001 for stage III & stage IV vs. stage I & stage II), histological type (OR = 2.563, 95% CI = 1.677–3.971, *P* < 0.001 for serous vs. endometrioid), and histologic grade (OR = 3.587, 95% CI = 2.501–5.184, *P* < 0.001 for G3 vs. G1&G2). Over-expressed CDCA8 was positively associated with clinical stage (OR = 2.252, 95% CI = 1.545–3.309, *P* < 0.001 for stage III & stage IV vs. stage I & stage II), histological type (OR = 3.573, 95% CI = 2.298–5.672, *P* < 0.001 for serous vs. endometrioid), and histologic grade (OR = 5.057, 95% CI = 3.486–7.418, *P* < 0.001 for G3 vs. G1&G2). Over-expressed NCAPG was positively associated with clinical stage (OR = 1.560, 95% CI = 1.077–2.268, *P* < 0.001 for stage III & stage IV vs. stage I & stage II), histological type (OR = 2.006, 95% CI = 1.322–3.072, *P*=0.001 for serous vs. endometrioid), and histologic grade (OR = 5.147, 95% CI = 3.549–7.546, *P* < 0.001 for G3 vs. G1&G2).

### 3.8. High Predictive Value of the Hub Genes for UCEC Diagnosis

**Figure 8:**
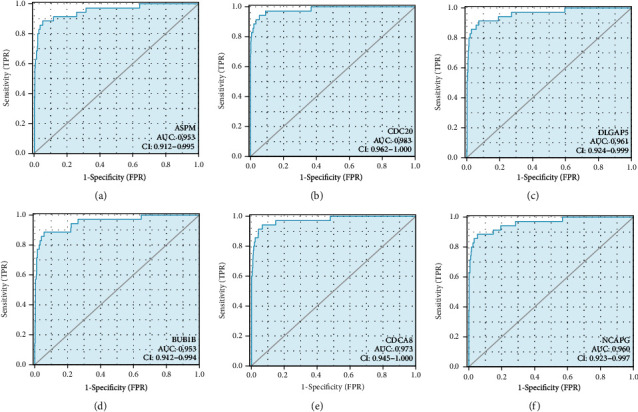
Receiver operating characteristic curve analysis to predict the diagnosis value of 6 hub genes for UCEC (a) ASPM. (b) CDC20. (c) DLGAP5. (d) BUB1B. (e) CDCA8. (f) NCAPG.

We used the ROC curve to analyze the discriminative power of the hub genes between UCEC tissues and normal tissues. The computed area under the curve (AUC) value ranged from 0.5 to 0.1. The closer AUC to 1, the better the diagnostic effect was. As shown in Figure 8, the AUC of the 6 hub genes was all above 0.95 (ASPM: AUC = 0.953, 95% CI = 0.912–0.995; CDC20: AUC = 0.983, 95% CI = 0.962–1.000; DLGAP5: AUC = 0.961, 95% CI = 0.924–0.999; BUB1B: AUC = 0.953, 95% CI = 0.912–0.994; CDCA8: AUC = 0.973, 95% CI = 0.945–1.000; NCAPG: AUC = 0.960, 95% CI = 0.923–0.997), suggesting that the 6 hub genes had significant diagnostic ability for UCEC.

### 3.9. Analysis of the Intrinsic Mechanisms of the 6 Hub Genes in UCEC

**Figure 9:**
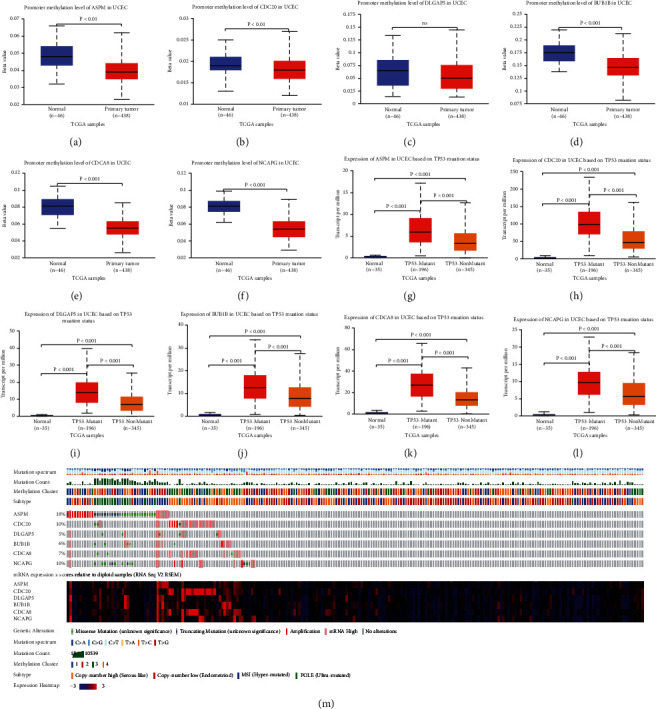
The underlying intrinsic pathogeneses of UCEC (a-f) The promoter methylation level of 6 hub genes is based on sample types. (g-l) The expression of 6 hub genes based on TP53 mutation status. (m) Genomic genetic variation in UCEC samples (*n* = 232). ns, *P* ≥ 0.05. UCEC, uterine corpus endometrial carcinoma.

First, we investigated the promoter methylation level of the 6 hub genes in 438 UCEC tissues and 46 normal tissues. We found that promoter methylation level of ASPM, CDC20, BUB1B, CDCA8, and NCAPG was observably lower in UCEC tissues than in normal tissues (*P* < 0.01) while DLGAP5 showed no significant statistical differences (Figures 9(a)–9(f)).

Next, as shown in Figures 9(g)–9(l), the expression of the 6 hub genes in UCEC TP53-mutant tissues (*n* = 196) was dramatically higher (*P* < 0.001) than in UCEC TP53-nonmutant tissues (*n* = 345) or normal tissues (*n* = 35), which indicated a close relationship between the hub genes expression and TP53-mutation status.

Lastly, by inputting the 6 hub genes into the cBioPortal website, we found that the genetic alterations of ASPM, CDC20, DLGAP5, BUB1B, CDCA8, and NCAPG among 232 UCEC samples were 18%, 10%, 5%, 6%, 7%, and 10%, respectively (Figure 9(m)). Among the 6 hub genes, ASPM was the most frequently altered gene. In particular, missense mutation, truncating mutation, and amplification were identified as the primary types of genetic alteration of ASPM in UCEC. It was worth noting that the types of UCEC samples with large mutation counts were mainly microsatellite instability-high or POLE-ultramutant types and the primary mutation spectrum type was *C* > *T* (Figure 9(m)).

### 3.10. Correlation between the Hub Genes Expression and Immune Infiltration

**Figure 10:**
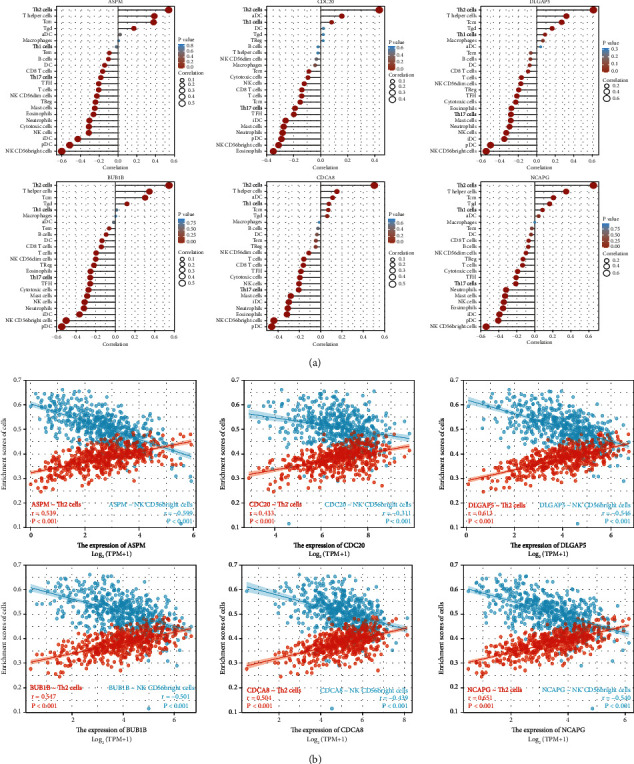
Correlation of immune infiltration and hub genes expression level in UCEC (a) The relationship between the relative abundance of 24 immune cells and hub genes expression level. (b) The relationship between Th2/NK CD56bright cells infiltration and hub genes expression level. UCEC, uterine corpus endometrial carcinoma.

Tumor-infiltrating immune cells were the primary components of the tumor microenvironment (TME) and had been considered to exert important effects on the progression of the tumor [32–34]. Therefore, we vividly presented the relationship between the hub gene expression and the level of infiltration of 24 immune cells in UCEC (Figure 10(a)).

As was shown, ASPM expression was positively correlated with the abundance of Th2 cells (*P* < 0.001), T helper cells (*P* < 0.001), Tcm (*P* < 0.001), Tgd (*P* < 0.001), and was negatively correlated with the abundance of NK CD56bright cells (*P* < 0.001), pDC (*P* < 0.001), iDC (*P* < 0.001), NK cells (*P* < 0.001), cytotoxic cells (*P* < 0.001), neutrophils (*P* < 0.001), eosinophils (*P* < 0.001), mast cells (*P* < 0.001), Treg (*P* < 0.001), NK CD56dim cells (*P* < 0.001), T cells (*P* < 0.001), Tfh (*P* < 0.001), Th17 cells (*P* < 0.001), CD8+ T cells (*P* < 0.001), DC (*P* < 0.001), B cells (*P*=0.017), Tem (*P*=0.029).

CDC20 expression was positively correlated with the abundance of Th2 cells (*P* < 0.001), aDC (*P* < 0.001) and was negatively correlated with the abundance of eosinophils (*P* < 0.001), NK CD56bright cells (*P* < 0.001), pDC (*P* < 0.001), neutrophils (*P* < 0.001), mast cells (*P* < 0.001), iDC (*P* < 0.001), Tfh (*P* < 0.001), Th17 cells (*P* < 0.001), Tcm (*P* < 0.001), T cells (*P* < 0.001), CD8+ T cells (*P* < 0.001), NK cells (*P*=0.004), cytotoxic cells (*P*=0.027), and Tem (*P*=0.041).

DLGAP5 expression was positively correlated with the abundance of Th2 cells (*P* < 0.001), T helper cells (*P* < 0.001), Tcm (*P* < 0.001), Tgd (*P* < 0.001), Th1 cells (*P*=0.035), and was negatively correlated with the abundance of NK CD56bright cells (*P* < 0.001), pDC (*P* < 0.001), iDC (*P* < 0.001), NK cells (*P* < 0.001), neutrophils (*P* < 0.001), mast cells (*P* < 0.001), Th17 cells (*P* < 0.001), eosinophils (*P* < 0.001), cytotoxic cells (*P* < 0.001), Tfh (*P* < 0.001), Treg (*P* < 0.001), NK CD56dim cells (*P* < 0.001), T cells (*P* < 0.001), and CD8+ T cells (*P*=0.029).

BUB1B expression was positively correlated with the abundance of Th2 cells (*P* < 0.001), T helper cells (*P* < 0.001), Tcm (*P* < 0.001), Tgd (*P*=0.005), and was negatively correlated with the abundance of pDC (*P* < 0.001), NK CD56bright cells (*P* < 0.001), iDC (*P* < 0.001), neutrophils (*P* < 0.001), NK cells (*P* < 0.001), mast cells (*P* < 0.001), cytotoxic cells (*P* < 0.001), Tfh (*P* < 0.001), Th17 cells (*P* < 0.001), eosinophils (*P* < 0.001), Treg (*P* < 0.001), NK CD56dim cells (*P* < 0.001), T cells (*P* < 0.001), CD8+ T cells (*P* < 0.001), DC (*P*=0.001), and B cells (*P*=0.027).

CDCA8 expression was positively correlated with the abundance of Th2 cells (*P* < 0.001), T helper cells (*P* < 0.001), aDC (*P*=0.008) and was negatively correlated with the abundance of pDC (*P* < 0.001), NK CD56bright cells (*P* < 0.001), iDC (*P* < 0.001), neutrophils (*P* < 0.001), eosinophils (*P* < 0.001), mast cells (*P* < 0.001), cytotoxic cells (*P* < 0.001), Tfh (*P* < 0.001), Th17 cells (*P* < 0.001), NK cells (*P* < 0.001), T cells (*P* < 0.001), CD8+ T cells (*P* < 0.001), and NK CD56dim cells (*P*=0.009).

NCAPG expression was positively correlated with the abundance of Th2 cells (*P* < 0.001), T helper cells (*P* < 0.001), Tcm (*P* < 0.001), Tgd (*P* < 0.001), Th1 cells (*P*=0.045) and was negatively correlated with the abundance of NK CD56bright cells (*P* < 0.001), pDC (*P* < 0.001), iDC (*P* < 0.001), eosinophils (*P* < 0.001), NK cells (*P* < 0.001), mast cells (*P* < 0.001), neutrophils (*P* < 0.001), Th17 cells (*P* < 0.001), Tfh (*P* < 0.001), cytotoxic cells (*P* < 0.001), T cells (*P*=0.001), Treg (*P*=0.002), and NK CD56dim cells (*P*=0.021).

The expression of the 6 hub genes was positively correlated with the abundance of Th2 cells and was all negatively correlated with the abundance of Th17 cells, NK CD56bright cells, pDC, NK cells, cytotoxic cells, neutrophils, eosinophils, mast cells, T cells, Tfh. We suspected that the above immunocytes might act as a regulator in the pathogenesis of the 6 hub genes. Since the 6 hub genes were all positively correlated with Th2 cells infiltration and negatively correlated with NK CD56bright cells infiltration, scatter plots about them were especially drawn in Figure 10(b).

### 3.11. The 6 Hub Genes Are Higher Expressed in CESC, UCS, And OV Tissues than in Corresponding Normal Tissues

According to the data from 306 CESC samples and corresponding 13 normal samples, the average mRNA expression level of the 6 hub genes was as follows: ASPM (Tumor: Normal = 3.74 ± 0.713 : 0.365 ± 0.663, *P* < 0.001); CDC20 (Tumor: Normal = 6.61 ± 0.637 : 1.17 ± 1.238, *P* < 0.001); DLGAP5 (Tumor: Normal = 4.038 ± 0.74 : 0.371 ± 0.724, *P* < 0.001); BUB1B (Tumor: Normal = 3.896 ± 0.753 : 0.609 ± 0.895, *P* < 0.001); CDCA8 (Tumor: Normal = 4.579 ± 0.688 : 0.947 ± 0.717, *P* < 0.001); NCAPG (Tumor: Normal = 3.828 ± 0.615 : 0.495 ± 0.817, *P* < 0.001).

According to the data from 57 UCS samples and corresponding 78 normal samples, the average mRNA expression level of the 6 hub genes was as follows: ASPM (Tumor: Normal = 3.29 ± 0.618 : 0.124 ± 0.173, *P* < 0.001); CDC20 (Tumor: Normal = 6.599 ± 0.776 : 0.785 ± 0.528, *P* < 0.001); DLGAP5 (Tumor: Normal = 3.746 ± 0.712 : 0.135 ± 0.21, *P* < 0.001); BUB1B (Tumor: Normal = 3.8 ± 0.618 : 0.533 ± 0.417, *P* < 0.001); CDCA8 (Tumor: Normal = 4.178 ± 0.612 : 0.82 ± 0.319, *P* < 0.001); NCAPG (Tumor: Normal = 3.703 ± 0.581 : 0.302 ± 0.295, *P* < 0.001).

According to the data from 427 OV samples and corresponding 88 normal samples, the average mRNA expression level of the 6 hub genes was as follows: ASPM (Tumor: Normal = 2.396 ± 0.772 : 0.203 ± 0.234, *P* < 0.001); CDC20 (Tumor: Normal = 5.831 ± 1.001 : 0.676 ± 0.539, *P* < 0.001); DLGAP5 (Tumor: Normal = 3 ± 0.897 : 0.224 ± 0.376, *P* < 0.001); BUB1B (Tumor: Normal = 2.921 ± 0.846 : 0.53 ± 0.368, *P* < 0.001); CDCA8 (Tumor: Normal = 3.827 ± 0.983 : 0.63 ± 0.318, *P* < 0.001); NCAPG (Tumor: Normal = 2.757 ± 0.853 : 0.332 ± 0.301, *P* < 0.001).

**Figure 11:**
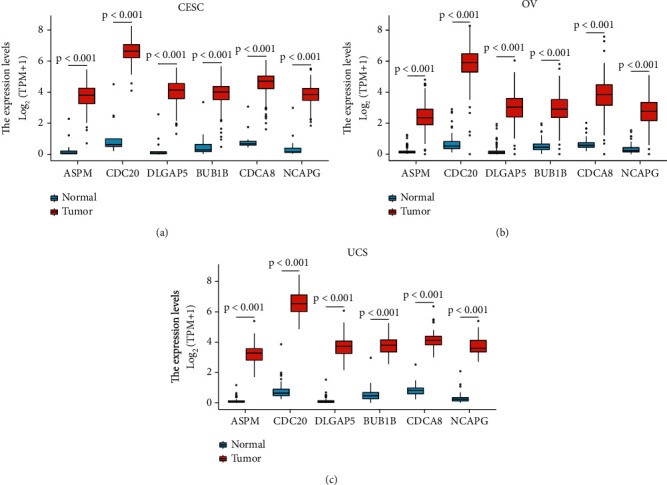
The comparison of hub genes mRNA expression level between tumor tissues and normal tissues in a female reproductive system based on UCSC Xena platform (a) CESC. (b) OV. (c) UCS. CESC, cervical squamous cell carcinoma and endocervical adenocarcinoma; OV, ovarian serous cystadenocarcinoma; UCS, uterine carcinosarcoma.

In general, the mRNA level of the above 6 hub genes was up-regulated in CESC, UCS, and OV tissues compared to corresponding normal tissue (Figure 11).

## 4. Discussion

In the present study, a total of 650 UCEC samples and 40 normal samples were collected from GSE17025, GSE63678, and TCGA-UCEC databases. Through analysis, we ended up with 125 co-up-regulated DEGs and 78 co-down-regulated DEGs. Under the GO/KEGG functional enrichment analysis, we found a plethora of DEGs were associated with the cell division process, for example, nuclear division, chromosome segregation, tubulin binding, microtubule motor activity, ATPase activity, and cell cycle. The results were consistent with the theory that uncontrolled DNA replication, abnormal proliferation, and dysregulated cell cycle control were essential molecular mechanisms in carcinogenesis [35].

By constructing the PPI network, we were excited to find out that more than 50 DEGs interacted with each other closely. This greatly aroused our curiosity and we hypothesized that those DEGs might be of paramount importance in UCEC tumorigenesis. Using the “Degree” method of the cytoHubba plugin tool, the top 10 nodes (including CDK1, ASPM, CCNB1, TOP2A, CDC20, DLGAP5, KIF11, BUB1B, CDCA8, and NCAPG) were located successfully and we considered them as hub genes for further research.

In the TCGA-UCEC database, we observed that mRNA expression of the 10 hub genes was significantly over-expressed in tumor tissues compared to normal tissues both in unpaired samples and paired samples. The same results were confirmed in the GEPIA database. Nevertheless, only partial hub genes over-expression was associated with an unfavorable prognosis. Compared with low gene expression, high expression of ASPM, BUB1B, CDCA8, and NCAPG was significantly correlated with a poor OS. In addition, high expression of ASPM, CDC20, DLGAP5, BUB1B, CDCA8, and NCAPG was markedly associated with a poor DSS. These results suggested that ASPM, CDC20, DLGAP5, BUB1B, CDCA8, and NCAPG might serve as biomarkers for poor prognosis in UCEC. Similarly, previous studies had stated that ASPM, CDC20, BUB1B, and CDCA8 expression could be potential poor survival prognostic biomarkers in lung adenocarcinoma [14], prostate/breast cancer [18,19], glioblastoma [12], respectively. Hence, we tried to conduct an appropriate, thorough, and in-depth understanding of the 6 hub genes in UCEC.

First, we explored the 6 hub genes' protein expression on the HPA website. Compared with normal tissues, we found that CDC20, DLGAP5, CDCA8, and NCAPG were higher staining in UCEC tissues. On account of lacking ASPM and BUB1B information, we then turned to the ULCAN-CPTAC platform for further validation. It was vividly shown that the protein expression level of CDC20, DLGAP5, BUB1B, CDCA8, and NCAPG was higher in UCEC tissues than in normal tissues. This was completely consistent with the result from HPA.

Next, we analyzed the association between the 6 hub genes and clinicopathological features. Notably, over-expressed expression of CDC20, DLGAP5, BUB1B, CDCA8, and NCAPG was all associated with poor prognostic clinicopathological characteristics (clinical stage, histological type, and histologic grade) by single gene logistics regression analysis while a little different was that over-expressed expression of ASPM was associated with age, histological type, and histologic grade. As tumor grade or stage increased, the mRNA expression of the 6 hub genes leaned to be higher. The above results suggested that the 6 hub genes functioned as oncogenes in UCEC. It should be mentioned that type I UCEC shared risk factors as exemplified by metabolic abnormalities such as obesity and diabetes [36]. Nonetheless, none of the expression of ASPM, CDC20, DLGAP5, BUB1B, CDCA8, and NCAPG was related to BMI and diabetes. Furthermore, the AUC of the 6 hub genes was all above 0.95. This strongly manifested a high discriminative power of the 6 hub genes for UCEC diagnosis between UCEC tissues and normal tissues.

Then, it was worth mentioning that the lower level of promoter methylation and the higher level of TP53-mutation were tied to the mechanisms of 5 (DLGAP5 excepted) or 6 hub genes in UCEC respectively. In terms of genetic alterations which encompassed missense mutation, truncating mutation, amplification, and mRNA high, ASPM was the most frequently altered gene in the 6 hub genes. We hypothesized that the contradictory trend of ASPM protein expression level might be related to its genetic alterations such as amplification and mutation. Gene expression was regulated in many ways. Transcriptional/post-transcriptional regulation and translational/post-translational regulation all played roles in the final protein expression [37]. Moreover, factors such as mRNA degradation and protein degradation might lead to the inconsistency between mRNA abundance and protein expression level [38].

UCEC therapy was not merely needed to keep a watchful eye on the intrinsic characteristics of UCEC cells but also needed to pay close attention to the dynamic communication with various components in its TME. It had been established that tumor-infiltrating immune cells played essential roles in the TME with their composition and distribution considered to be linked with tumorigenesis and development [32–34]. We discovered that the expression of the 6 hub genes was positively correlated with the abundance of Th2 cells, and was all negatively correlated with the abundance of Th17 cells, NK CD56bright cells, pDC, NK cells, cytotoxic cells, neutrophils, eosinophils, mast cells, T cells, Tfh. Indeed, there were limited studies on the association between the 6 hub genes and immune infiltrations. James et al observed that enhanced expansion of Treg cells was accompanied by elevated expression of CDC20 and inflammatory tissue migratory markers (ITGA4, CXCR1) [39]. Similarly, Seon et al found that responses resulting from hypoxic stress, including upregulation of CDC20, were accountable for the superior expansion of NK cells via ERK/STAT3 activation in patients with advanced cancer [40]. A previous study stated that both chemokines (CXCL12, IP-10, and CCL27) and cytokines profiles (IL-1*β* and IL-6) in the TME suppressed NK cells (major anti-tumoral effector cells) expression and function, then promoting UCEC progression [41]. It is important to understand how the 6 hub genes interact with their surrounding infiltrating immune cells during oncogenesis. Once the underlying immune-related mechanisms were clarified by the experimental method, the 6 hub genes might be useful for novel immunotherapy.

Finally, we explored the role of the 6 hub genes in the female reproductive system. Intriguingly, the mRNA level of the 6 hub genes was up-regulated in CESC, UCS, and OV tissues compared to corresponding normal tissues. The results demonstrated that the 6 hub genes might play a pivotal role in the tumorigenesis of the female reproductive system and its verification could shed some light on the current research.

However, there are still deficiencies in this study. Clinical samples may need to collect to validate the results. Further experimental verifications are needed to dissect more carefully the biological functions of the 6 hub genes in vitro and in vivo.

## 5. Conclusions

ASPM, CDC20, DLGAP5, BUB1B, CDCA8, and NCAPG may be considered diagnostic and prognostic biomarkers for UCEC. Promoter methylation level, TP53-mutation status, genomic genetic variation, and immune infiltration were involved in the UCEC pathogenesis of the 6 hub genes.

## Figures and Tables

**Table 1:** Top 15 GO functional enrichment of DEGs. GO, gene ontology; DEGs, differentially expressed genes; BP, biological process; CC, cellular component; MF, molecular function.

Ontology	ID	Description	GeneRatio	p.adjust	*Q* value	Count
BP	GO:0000280	Nuclear division	39/188	4.71026E-24	4.09529E-24	39
BP	GO:0048285	Organelle fission	39/188	1.11662E-22	9.70834E-23	39
BP	GO:0140014	Mitotic nuclear division	35/188	1.09717E-25	9.53925E-26	35
BP	GO:0007059	Chromosome segregation	31/188	4.91768E-19	4.27563E-19	31
BP	GO:0000819	Sister chromatid segregation	29/188	3.07213E-23	2.67104E-23	29
CC	GO:0005819	Spindle	30/192	1.50391E-17	1.26878E-17	30
CC	GO:0098687	Chromosomal region	21/192	1.73049E-09	1.45993E-09	21
CC	GO:0000793	Condensed chromosome	19/192	9.20435E-11	7.76528E-11	19
CC	GO:0005874	Microtubule	18/192	2.59157E-06	2.18639E-06	18
CC	GO:0000775	Chromosome, centromeric region	17/192	4.6304E-10	3.90646E-10	17
MF	GO:0016887	ATPase activity	17/188	1.09773E-4	1.01309E-4	17
MF	GO:0015631	Tubulin binding	16/188	1.09773E-4	1.01309E-4	16
MF	GO:0008017	Microtubule binding	13/188	2.02801E-4	1.87163E-4	13
MF	GO:0003777	Microtubule motor activity	10/188	8.6576E-06	7.99E-06	10
MF	GO:0003774	Motor activity	10/188	2.02801E-4	1.87163E-4	10

**Table 2:** KEGG functional enrichment analysis of DEGs. KEGG, kyoto encyclopedia of genes and genomes.

Ontology	ID	Description	GeneRatio	p.adjust	Qvalue	Count
KEGG	hsa04110	Cell cycle	12/89	1.58E-06	1.48E-06	12
KEGG	hsa04114	Oocyte meiosis	9/89	9.96E-04	9.32E-04	9
KEGG	hsa04115	p53 signaling pathway	5/89	0.073	0.068	5
KEGG	hsa00270	Cysteine and methionine metabolism	4/89	0.097	0.091	4

**Table 3:** Top 10 nodes ranked by the “Degree” method in the protein-protein interaction network.

Rank	Name	Score
1	CDK1	116
2	ASPM	96
3	CCNB1	94
3	TOP2A	94
5	CDC20	92
6	DLGAP5	90
6	KIF11	90
8	BUB1B	88
9	CDCA8	86
10	NCAPG	84

## Data Availability

Datasets used to support the findings of the study can be obtained from the corresponding author upon request.

## Supplementary Materials

Supplementary Materials See Tables S1-S2 in the Supplementary Material. Table S1. Clinicopathological characteristics of UCEC patients with differential ASPM/CDC20/DLGAP5/BUB1B/CDCA8/NCAPG expression. UCEC, uterine corpus endometrial carcinoma. Table S2. Association of ASPM/CDC20/DLGAP5/BUB1B/CDCA8/NCAPG expression with clinicopathological characteristics in UCEC patients (single gene logistics regression analysis). UCEC, uterine corpus endometrial carcinoma.
