# Supplementary material for: ASPM, CDC20, DLGAP5, BUB1B, CDCA8, and NCAPG May Serve as Diagnostic and Prognostic Biomarkers in Endometrial Carcinoma
Source: Genet Res (Camb). 2022 Sep 17;2022:3217248. doi: 10.1155/2022/3217248 (PMC9509287; doi:10.1155/2022/3217248)
Supplement: Supplementary Materials — See Tables S1-S2 in the Supplementary Material. Table S1. Clinicopathological characteristics of UCEC patients with differential ASPM/CDC20/DLGAP5/BUB1B/CDCA8/NCAPG expression. UCEC, uterine corpus endometrial carcinoma. Table S2. Association of ASPM/CDC20/DLGAP5/BUB1B/CDCA8/NCAPG expression with clinicopathological characteristics in UCEC patients (single gene logistics regression analysis). UCEC, uterine corpus endometrial carcinoma. [file 3217248.f1.zip › 3217248.f1/Table S1 (1).docx]

| Characteristic | Low expression of ASPM | High expression of ASPM | p |
| --- | --- | --- | --- |
| n | 276 | 276 |  |
| Clinical stage, n (%) |  |  | 0.195 |
| Stage I | 183 (33.2%) | 159 (28.8%) |  |
| Stage II | 23 (4.2%) | 28 (5.1%) |  |
| Stage III | 56 (10.1%) | 74 (13.4%) |  |
| Stage IV | 14 (2.5%) | 15 (2.7%) |  |
| Primary therapy outcome, n (%) |  |  | 0.400 |
| PD | 8 (1.7%) | 12 (2.5%) |  |
| SD | 3 (0.6%) | 3 (0.6%) |  |
| PR | 4 (0.8%) | 8 (1.7%) |  |
| CR | 234 (48.8%) | 208 (43.3%) |  |
| Race, n (%) |  |  | 0.047 |
| Asian | 13 (2.6%) | 7 (1.4%) |  |
| Black or African American | 44 (8.7%) | 64 (12.6%) |  |
| White | 197 (38.9%) | 182 (35.9%) |  |
| Age, n (%) |  |  | 0.016 |
| <=60 | 117 (21.3%) | 89 (16.2%) |  |
| >60 | 157 (28.6%) | 186 (33.9%) |  |
| Weight, n (%) |  |  | 0.014 |
| <=80 | 106 (20.1%) | 137 (25.9%) |  |
| >80 | 156 (29.5%) | 129 (24.4%) |  |
| Height, n (%) |  |  | 0.446 |
| <=160 | 117 (22.4%) | 130 (24.9%) |  |
| >160 | 141 (27%) | 135 (25.8%) |  |
| BMI, n (%) |  |  | 0.182 |
| <=30 | 97 (18.7%) | 115 (22.2%) |  |
| >30 | 160 (30.8%) | 147 (28.3%) |  |
| Histological type, n (%) |  |  | < 0.001 |
| Endometrioid | 230 (41.7%) | 180 (32.6%) |  |
| Mixed | 7 (1.3%) | 17 (3.1%) |  |
| Serous | 39 (7.1%) | 79 (14.3%) |  |
| Residual tumor, n (%) |  |  | 0.557 |
| R0 | 192 (46.5%) | 183 (44.3%) |  |
| R1 | 9 (2.2%) | 13 (3.1%) |  |
| R2 | 7 (1.7%) | 9 (2.2%) |  |
| Histologic grade, n (%) |  |  | < 0.001 |
| G1 | 77 (14.2%) | 21 (3.9%) |  |
| G2 | 74 (13.7%) | 46 (8.5%) |  |
| G3 | 120 (22.2%) | 203 (37.5%) |  |
| Tumor invasion(%), n (%) |  |  | 0.208 |
| <50 | 145 (30.6%) | 114 (24.1%) |  |
| >=50 | 107 (22.6%) | 108 (22.8%) |  |
| Menopause status, n (%) |  |  | 0.381 |
| Pre | 21 (4.2%) | 14 (2.8%) |  |
| Peri | 7 (1.4%) | 10 (2%) |  |
| Post | 226 (44.7%) | 228 (45.1%) |  |
| Hormones therapy, n (%) |  |  | 0.325 |
| No | 153 (44.5%) | 144 (41.9%) |  |
| Yes | 20 (5.8%) | 27 (7.8%) |  |
| Diabetes, n (%) |  |  | 0.795 |
| No | 169 (37.5%) | 159 (35.3%) |  |
| Yes | 61 (13.5%) | 62 (13.7%) |  |
| Radiation therapy, n (%) |  |  | 0.093 |
| No | 152 (28.8%) | 127 (24.1%) |  |
| Yes | 116 (22%) | 132 (25%) |  |
| Surgical approach, n (%) |  |  | 0.250 |
| Minimally Invasive | 99 (18.7%) | 109 (20.6%) |  |
| open | 171 (32.3%) | 151 (28.5%) |  |
| OS event, n (%) |  |  | 0.054 |
| Alive | 238 (43.1%) | 220 (39.9%) |  |
| Dead | 38 (6.9%) | 56 (10.1%) |  |
| DSS event, n (%) |  |  | 0.032 |
| Alive | 252 (45.8%) | 235 (42.7%) |  |
| Dead | 23 (4.2%) | 40 (7.3%) |  |
| PFI event, n (%) |  |  | 0.005 |
| Alive | 226 (40.9%) | 197 (35.7%) |  |
| Dead | 50 (9.1%) | 79 (14.3%) |  |
| Age, meidan (IQR) | 63 (56.25, 70.75) | 65 (58, 72) | 0.028 |

**Table S1.1.** Clinicopathological characteristics of UCEC patients with differential ASPM expression. UCEC, uterine corpus endometrial carcinoma.

| Characteristic | Low expression of CDC20 | High expression of CDC20 | p |
| --- | --- | --- | --- |
| n | 276 | 276 |  |
| Clinical stage, n (%) |  |  | 0.009 |
| Stage I | 189 (34.2%) | 153 (27.7%) |  |
| Stage II | 25 (4.5%) | 26 (4.7%) |  |
| Stage III | 50 (9.1%) | 80 (14.5%) |  |
| Stage IV | 12 (2.2%) | 17 (3.1%) |  |
| Primary therapy outcome, n (%) |  |  | 0.184 |
| PD | 6 (1.2%) | 14 (2.9%) |  |
| SD | 2 (0.4%) | 4 (0.8%) |  |
| PR | 6 (1.2%) | 6 (1.2%) |  |
| CR | 232 (48.3%) | 210 (43.8%) |  |
| Race, n (%) |  |  | 0.641 |
| Asian | 11 (2.2%) | 9 (1.8%) |  |
| Black or African American | 51 (10.1%) | 57 (11.2%) |  |
| White | 197 (38.9%) | 182 (35.9%) |  |
| Age, n (%) |  |  | 0.349 |
| <=60 | 109 (19.9%) | 97 (17.7%) |  |
| >60 | 166 (30.2%) | 177 (32.2%) |  |
| Weight, n (%) |  |  | 0.136 |
| <=80 | 112 (21.2%) | 131 (24.8%) |  |
| >80 | 151 (28.6%) | 134 (25.4%) |  |
| Height, n (%) |  |  | 0.359 |
| <=160 | 118 (22.6%) | 129 (24.7%) |  |
| >160 | 144 (27.5%) | 132 (25.2%) |  |
| BMI, n (%) |  |  | 0.231 |
| <=30 | 99 (19.1%) | 113 (21.8%) |  |
| >30 | 161 (31%) | 146 (28.1%) |  |
| Histological type, n (%) |  |  | < 0.001 |
| Endometrioid | 236 (42.8%) | 174 (31.5%) |  |
| Mixed | 12 (2.2%) | 12 (2.2%) |  |
| Serous | 28 (5.1%) | 90 (16.3%) |  |
| Residual tumor, n (%) |  |  | 0.422 |
| R0 | 188 (45.5%) | 187 (45.3%) |  |
| R1 | 13 (3.1%) | 9 (2.2%) |  |
| R2 | 6 (1.5%) | 10 (2.4%) |  |
| Histologic grade, n (%) |  |  | < 0.001 |
| G1 | 84 (15.5%) | 14 (2.6%) |  |
| G2 | 80 (14.8%) | 40 (7.4%) |  |
| G3 | 109 (20.1%) | 214 (39.6%) |  |
| Tumor invasion(%), n (%) |  |  | 0.140 |
| <50 | 144 (30.4%) | 115 (24.3%) |  |
| >=50 | 104 (21.9%) | 111 (23.4%) |  |
| Menopause status, n (%) |  |  | 0.449 |
| Pre | 21 (4.2%) | 14 (2.8%) |  |
| Peri | 9 (1.8%) | 8 (1.6%) |  |
| Post | 223 (44.1%) | 231 (45.7%) |  |
| Hormones therapy, n (%) |  |  | 0.304 |
| No | 143 (41.6%) | 154 (44.8%) |  |
| Yes | 27 (7.8%) | 20 (5.8%) |  |
| Diabetes, n (%) |  |  | 0.532 |
| No | 158 (35%) | 170 (37.7%) |  |
| Yes | 64 (14.2%) | 59 (13.1%) |  |
| Radiation therapy, n (%) |  |  | 0.003 |
| No | 160 (30.4%) | 119 (22.6%) |  |
| Yes | 109 (20.7%) | 139 (26.4%) |  |
| Surgical approach, n (%) |  |  | 0.294 |
| Minimally Invasive | 98 (18.5%) | 110 (20.8%) |  |
| open | 168 (31.7%) | 154 (29.1%) |  |
| OS event, n (%) |  |  | 0.089 |
| Alive | 237 (42.9%) | 221 (40%) |  |
| Dead | 39 (7.1%) | 55 (10%) |  |
| DSS event, n (%) |  |  | 0.032 |
| Alive | 252 (45.8%) | 235 (42.7%) |  |
| Dead | 23 (4.2%) | 40 (7.3%) |  |
| PFI event, n (%) |  |  | 0.005 |
| Alive | 226 (40.9%) | 197 (35.7%) |  |
| Dead | 50 (9.1%) | 79 (14.3%) |  |
| Age, meidan (IQR) | 63 (56, 71.5) | 64.5 (57.25, 71) | 0.290 |

**Table S1.2.** Clinicopathological characteristics of UCEC patients with differential CDC20 expression. UCEC, uterine corpus endometrial carcinoma.

| Characteristic | Low expression of DLGAP5 | High expression of DLGAP5 | p |
| --- | --- | --- | --- |
| n | 276 | 276 |  |
| Clinical stage, n (%) |  |  | < 0.001 |
| Stage I | 199 (36.1%) | 143 (25.9%) |  |
| Stage II | 21 (3.8%) | 30 (5.4%) |  |
| Stage III | 46 (8.3%) | 84 (15.2%) |  |
| Stage IV | 10 (1.8%) | 19 (3.4%) |  |
| Primary therapy outcome, n (%) |  |  | 0.176 |
| PD | 8 (1.7%) | 12 (2.5%) |  |
| SD | 3 (0.6%) | 3 (0.6%) |  |
| PR | 3 (0.6%) | 9 (1.9%) |  |
| CR | 235 (49%) | 207 (43.1%) |  |
| Race, n (%) |  |  | 0.311 |
| Asian | 10 (2%) | 10 (2%) |  |
| Black or African American | 48 (9.5%) | 60 (11.8%) |  |
| White | 200 (39.4%) | 179 (35.3%) |  |
| Age, n (%) |  |  | 0.447 |
| <=60 | 108 (19.7%) | 98 (17.9%) |  |
| >60 | 167 (30.4%) | 176 (32.1%) |  |
| Weight, n (%) |  |  | 0.037 |
| <=80 | 110 (20.8%) | 133 (25.2%) |  |
| >80 | 156 (29.5%) | 129 (24.4%) |  |
| Height, n (%) |  |  | 0.106 |
| <=160 | 114 (21.8%) | 133 (25.4%) |  |
| >160 | 148 (28.3%) | 128 (24.5%) |  |
| BMI, n (%) |  |  | 0.786 |
| <=30 | 105 (20.2%) | 107 (20.6%) |  |
| >30 | 157 (30.3%) | 150 (28.9%) |  |
| Histological type, n (%) |  |  | < 0.001 |
| Endometrioid | 233 (42.2%) | 177 (32.1%) |  |
| Mixed | 11 (2%) | 13 (2.4%) |  |
| Serous | 32 (5.8%) | 86 (15.6%) |  |
| Residual tumor, n (%) |  |  | 0.957 |
| R0 | 197 (47.7%) | 178 (43.1%) |  |
| R1 | 11 (2.7%) | 11 (2.7%) |  |
| R2 | 8 (1.9%) | 8 (1.9%) |  |
| Histologic grade, n (%) |  |  | < 0.001 |
| G1 | 80 (14.8%) | 18 (3.3%) |  |
| G2 | 83 (15.3%) | 37 (6.8%) |  |
| G3 | 110 (20.3%) | 213 (39.4%) |  |
| Tumor invasion(%), n (%) |  |  | 0.214 |
| <50 | 146 (30.8%) | 113 (23.8%) |  |
| >=50 | 108 (22.8%) | 107 (22.6%) |  |
| Menopause status, n (%) |  |  | 0.442 |
| Pre | 18 (3.6%) | 17 (3.4%) |  |
| Peri | 11 (2.2%) | 6 (1.2%) |  |
| Post | 223 (44.1%) | 231 (45.7%) |  |
| Hormones therapy, n (%) |  |  | 0.441 |
| No | 149 (43.3%) | 148 (43%) |  |
| Yes | 27 (7.8%) | 20 (5.8%) |  |
| Diabetes, n (%) |  |  | 0.916 |
| No | 169 (37.5%) | 159 (35.3%) |  |
| Yes | 62 (13.7%) | 61 (13.5%) |  |
| Radiation therapy, n (%) |  |  | 0.011 |
| No | 157 (29.8%) | 122 (23.1%) |  |
| Yes | 111 (21.1%) | 137 (26%) |  |
| Surgical approach, n (%) |  |  | 0.123 |
| Minimally Invasive | 96 (18.1%) | 112 (21.1%) |  |
| open | 172 (32.5%) | 150 (28.3%) |  |
| OS event, n (%) |  |  | 0.141 |
| Alive | 236 (42.8%) | 222 (40.2%) |  |
| Dead | 40 (7.2%) | 54 (9.8%) |  |
| DSS event, n (%) |  |  | 0.032 |
| Alive | 252 (45.8%) | 235 (42.7%) |  |
| Dead | 23 (4.2%) | 40 (7.3%) |  |
| PFI event, n (%) |  |  | 0.009 |
| Alive | 225 (40.8%) | 198 (35.9%) |  |
| Dead | 51 (9.2%) | 78 (14.1%) |  |
| Age, mean ± SD | 63.71 ± 11.3 | 64.45 ± 10.84 | 0.430 |

**Table S1.3.** Clinicopathological characteristics of UCEC patients with differential DLGAP5 expression. UCEC, uterine corpus endometrial carcinoma.

| Characteristic | Low expression of BUB1B | High expression of BUB1B | p |
| --- | --- | --- | --- |
| n | 276 | 276 |  |
| Clinical stage, n (%) |  |  | 0.002 |
| Stage I | 192 (34.8%) | 150 (27.2%) |  |
| Stage II | 24 (4.3%) | 27 (4.9%) |  |
| Stage III | 48 (8.7%) | 82 (14.9%) |  |
| Stage IV | 12 (2.2%) | 17 (3.1%) |  |
| Primary therapy outcome, n (%) |  |  | 0.568 |
| PD | 9 (1.9%) | 11 (2.3%) |  |
| SD | 3 (0.6%) | 3 (0.6%) |  |
| PR | 4 (0.8%) | 8 (1.7%) |  |
| CR | 232 (48.3%) | 210 (43.8%) |  |
| Race, n (%) |  |  | 0.320 |
| Asian | 9 (1.8%) | 11 (2.2%) |  |
| Black or African American | 48 (9.5%) | 60 (11.8%) |  |
| White | 198 (39.1%) | 181 (35.7%) |  |
| Age, n (%) |  |  | 0.447 |
| <=60 | 108 (19.7%) | 98 (17.9%) |  |
| >60 | 167 (30.4%) | 176 (32.1%) |  |
| Weight, n (%) |  |  | 0.053 |
| <=80 | 109 (20.6%) | 134 (25.4%) |  |
| >80 | 153 (29%) | 132 (25%) |  |
| Height, n (%) |  |  | 0.349 |
| <=160 | 116 (22.2%) | 131 (25%) |  |
| >160 | 142 (27.2%) | 134 (25.6%) |  |
| BMI, n (%) |  |  | 0.328 |
| <=30 | 99 (19.1%) | 113 (21.8%) |  |
| >30 | 158 (30.4%) | 149 (28.7%) |  |
| Histological type, n (%) |  |  | < 0.001 |
| Endometrioid | 229 (41.5%) | 181 (32.8%) |  |
| Mixed | 8 (1.4%) | 16 (2.9%) |  |
| Serous | 39 (7.1%) | 79 (14.3%) |  |
| Residual tumor, n (%) |  |  | 0.684 |
| R0 | 194 (47%) | 181 (43.8%) |  |
| R1 | 12 (2.9%) | 10 (2.4%) |  |
| R2 | 10 (2.4%) | 6 (1.5%) |  |
| Histologic grade, n (%) |  |  | < 0.001 |
| G1 | 73 (13.5%) | 25 (4.6%) |  |
| G2 | 77 (14.2%) | 43 (7.9%) |  |
| G3 | 123 (22.7%) | 200 (37%) |  |
| Tumor invasion(%), n (%) |  |  | 0.374 |
| <50 | 143 (30.2%) | 116 (24.5%) |  |
| >=50 | 109 (23%) | 106 (22.4%) |  |
| Menopause status, n (%) |  |  | 0.649 |
| Pre | 19 (3.8%) | 16 (3.2%) |  |
| Peri | 10 (2%) | 7 (1.4%) |  |
| Post | 224 (44.3%) | 230 (45.5%) |  |
| Hormones therapy, n (%) |  |  | 0.679 |
| No | 151 (43.9%) | 146 (42.4%) |  |
| Yes | 26 (7.6%) | 21 (6.1%) |  |
| Diabetes, n (%) |  |  | 0.992 |
| No | 166 (36.8%) | 162 (35.9%) |  |
| Yes | 63 (14%) | 60 (13.3%) |  |
| Radiation therapy, n (%) |  |  | 0.248 |
| No | 149 (28.3%) | 130 (24.7%) |  |
| Yes | 119 (22.6%) | 129 (24.5%) |  |
| Surgical approach, n (%) |  |  | 0.263 |
| Minimally Invasive | 98 (18.5%) | 110 (20.8%) |  |
| open | 169 (31.9%) | 153 (28.9%) |  |
| OS event, n (%) |  |  | 0.031 |
| Alive | 239 (43.3%) | 219 (39.7%) |  |
| Dead | 37 (6.7%) | 57 (10.3%) |  |
| DSS event, n (%) |  |  | 0.102 |
| Alive | 251 (45.6%) | 236 (42.9%) |  |
| Dead | 25 (4.5%) | 38 (6.9%) |  |
| PFI event, n (%) |  |  | 0.070 |
| Alive | 221 (40%) | 202 (36.6%) |  |
| Dead | 55 (10%) | 74 (13.4%) |  |
| Age, meidan (IQR) | 63 (57, 72) | 64 (57, 71) | 0.486 |

**Table S1.4.** Clinicopathological characteristics of UCEC patients with differential BUB1B expression. UCEC, uterine corpus endometrial carcinoma.

| Characteristic | Low expression of CDCA8 | High expression of CDCA8 | p |
| --- | --- | --- | --- |
| n | 276 | 276 |  |
| Clinical stage, n (%) |  |  | < 0.001 |
| Stage I | 193 (35%) | 149 (27%) |  |
| Stage II | 26 (4.7%) | 25 (4.5%) |  |
| Stage III | 48 (8.7%) | 82 (14.9%) |  |
| Stage IV | 9 (1.6%) | 20 (3.6%) |  |
| Primary therapy outcome, n (%) |  |  | 0.265 |
| PD | 9 (1.9%) | 11 (2.3%) |  |
| SD | 3 (0.6%) | 3 (0.6%) |  |
| PR | 3 (0.6%) | 9 (1.9%) |  |
| CR | 232 (48.3%) | 210 (43.8%) |  |
| Race, n (%) |  |  | 0.640 |
| Asian | 10 (2%) | 10 (2%) |  |
| Black or African American | 50 (9.9%) | 58 (11.4%) |  |
| White | 195 (38.5%) | 184 (36.3%) |  |
| Age, n (%) |  |  | 0.088 |
| <=60 | 113 (20.6%) | 93 (16.9%) |  |
| >60 | 161 (29.3%) | 182 (33.2%) |  |
| Weight, n (%) |  |  | 0.011 |
| <=80 | 105 (19.9%) | 138 (26.1%) |  |
| >80 | 156 (29.5%) | 129 (24.4%) |  |
| Height, n (%) |  |  | 0.399 |
| <=160 | 117 (22.4%) | 130 (24.9%) |  |
| >160 | 142 (27.2%) | 134 (25.6%) |  |
| BMI, n (%) |  |  | 0.061 |
| <=30 | 94 (18.1%) | 118 (22.7%) |  |
| >30 | 163 (31.4%) | 144 (27.7%) |  |
| Histological type, n (%) |  |  | < 0.001 |
| Endometrioid | 234 (42.4%) | 176 (31.9%) |  |
| Mixed | 10 (1.8%) | 14 (2.5%) |  |
| Serous | 32 (5.8%) | 86 (15.6%) |  |
| Residual tumor, n (%) |  |  | 0.780 |
| R0 | 190 (46%) | 185 (44.8%) |  |
| R1 | 10 (2.4%) | 12 (2.9%) |  |
| R2 | 7 (1.7%) | 9 (2.2%) |  |
| Histologic grade, n (%) |  |  | < 0.001 |
| G1 | 81 (15%) | 17 (3.1%) |  |
| G2 | 79 (14.6%) | 41 (7.6%) |  |
| G3 | 114 (21.1%) | 209 (38.6%) |  |
| Tumor invasion(%), n (%) |  |  | 0.144 |
| <50 | 145 (30.6%) | 114 (24.1%) |  |
| >=50 | 105 (22.2%) | 110 (23.2%) |  |
| Menopause status, n (%) |  |  | 0.961 |
| Pre | 18 (3.6%) | 17 (3.4%) |  |
| Peri | 9 (1.8%) | 8 (1.6%) |  |
| Post | 227 (44.9%) | 227 (44.9%) |  |
| Hormones therapy, n (%) |  |  | 0.449 |
| No | 143 (41.6%) | 154 (44.8%) |  |
| Yes | 26 (7.6%) | 21 (6.1%) |  |
| Diabetes, n (%) |  |  | 1.000 |
| No | 166 (36.8%) | 162 (35.9%) |  |
| Yes | 62 (13.7%) | 61 (13.5%) |  |
| Radiation therapy, n (%) |  |  | 0.022 |
| No | 155 (29.4%) | 124 (23.5%) |  |
| Yes | 112 (21.3%) | 136 (25.8%) |  |
| Surgical approach, n (%) |  |  | 0.446 |
| Minimally Invasive | 100 (18.9%) | 108 (20.4%) |  |
| open | 167 (31.5%) | 155 (29.2%) |  |
| OS event, n (%) |  |  | 0.031 |
| Alive | 239 (43.3%) | 219 (39.7%) |  |
| Dead | 37 (6.7%) | 57 (10.3%) |  |
| DSS event, n (%) |  |  | 0.032 |
| Alive | 252 (45.8%) | 235 (42.7%) |  |
| Dead | 23 (4.2%) | 40 (7.3%) |  |
| PFI event, n (%) |  |  | 0.005 |
| Alive | 226 (40.9%) | 197 (35.7%) |  |
| Dead | 50 (9.1%) | 79 (14.3%) |  |
| Age, meidan (IQR) | 63 (56, 71) | 64 (58, 71) | 0.137 |

**Table S1.5.** Clinicopathological characteristics of UCEC patients with differential CDCA8 expression. UCEC, uterine corpus endometrial carcinoma.

| Characteristic | Low expression of NCAPG | High expression of NCAPG | p |
| --- | --- | --- | --- |
| n | 276 | 276 |  |
| Clinical stage, n (%) |  |  | 0.070 |
| Stage I | 186 (33.7%) | 156 (28.3%) |  |
| Stage II | 23 (4.2%) | 28 (5.1%) |  |
| Stage III | 55 (10%) | 75 (13.6%) |  |
| Stage IV | 12 (2.2%) | 17 (3.1%) |  |
| Primary therapy outcome, n (%) |  |  | 0.749 |
| PD | 9 (1.9%) | 11 (2.3%) |  |
| SD | 3 (0.6%) | 3 (0.6%) |  |
| PR | 5 (1%) | 7 (1.5%) |  |
| CR | 235 (49%) | 207 (43.1%) |  |
| Race, n (%) |  |  | 0.063 |
| Asian | 10 (2%) | 10 (2%) |  |
| Black or African American | 44 (8.7%) | 64 (12.6%) |  |
| White | 203 (40%) | 176 (34.7%) |  |
| Age, n (%) |  |  | 0.488 |
| <=60 | 108 (19.7%) | 98 (17.9%) |  |
| >60 | 168 (30.6%) | 175 (31.9%) |  |
| Weight, n (%) |  |  | 0.096 |
| <=80 | 111 (21%) | 132 (25%) |  |
| >80 | 152 (28.8%) | 133 (25.2%) |  |
| Height, n (%) |  |  | 0.205 |
| <=160 | 116 (22.2%) | 131 (25%) |  |
| >160 | 146 (27.9%) | 130 (24.9%) |  |
| BMI, n (%) |  |  | 0.275 |
| <=30 | 100 (19.3%) | 112 (21.6%) |  |
| >30 | 161 (31%) | 146 (28.1%) |  |
| Histological type, n (%) |  |  | 0.002 |
| Endometrioid | 223 (40.4%) | 187 (33.9%) |  |
| Mixed | 9 (1.6%) | 15 (2.7%) |  |
| Serous | 44 (8%) | 74 (13.4%) |  |
| Residual tumor, n (%) |  |  | 0.763 |
| R0 | 191 (46.2%) | 184 (44.6%) |  |
| R1 | 10 (2.4%) | 12 (2.9%) |  |
| R2 | 7 (1.7%) | 9 (2.2%) |  |
| Histologic grade, n (%) |  |  | < 0.001 |
| G1 | 80 (14.8%) | 18 (3.3%) |  |
| G2 | 79 (14.6%) | 41 (7.6%) |  |
| G3 | 111 (20.5%) | 212 (39.2%) |  |
| Tumor invasion(%), n (%) |  |  | 0.323 |
| <50 | 143 (30.2%) | 116 (24.5%) |  |
| >=50 | 108 (22.8%) | 107 (22.6%) |  |
| Menopause status, n (%) |  |  | 0.746 |
| Pre | 17 (3.4%) | 18 (3.6%) |  |
| Peri | 10 (2%) | 7 (1.4%) |  |
| Post | 225 (44.5%) | 229 (45.3%) |  |
| Hormones therapy, n (%) |  |  | 0.242 |
| No | 146 (42.4%) | 151 (43.9%) |  |
| Yes | 28 (8.1%) | 19 (5.5%) |  |
| Diabetes, n (%) |  |  | 0.825 |
| No | 165 (36.6%) | 163 (36.1%) |  |
| Yes | 64 (14.2%) | 59 (13.1%) |  |
| Radiation therapy, n (%) |  |  | 0.029 |
| No | 157 (29.8%) | 122 (23.1%) |  |
| Yes | 115 (21.8%) | 133 (25.2%) |  |
| Surgical approach, n (%) |  |  | 0.684 |
| Minimally Invasive | 102 (19.2%) | 106 (20%) |  |
| open | 165 (31.1%) | 157 (29.6%) |  |
| OS event, n (%) |  |  | 0.031 |
| Alive | 239 (43.3%) | 219 (39.7%) |  |
| Dead | 37 (6.7%) | 57 (10.3%) |  |
| DSS event, n (%) |  |  | 0.030 |
| Alive | 253 (46%) | 234 (42.5%) |  |
| Dead | 23 (4.2%) | 40 (7.3%) |  |
| PFI event, n (%) |  |  | 0.016 |
| Alive | 224 (40.6%) | 199 (36.1%) |  |
| Dead | 52 (9.4%) | 77 (13.9%) |  |
| Age, mean ± SD | 63.83 ± 11.28 | 64.33 ± 10.86 | 0.597 |

**Table S1.6.** Clinicopathological characteristics of UCEC patients with differential NCAPG expression. UCEC, uterine corpus endometrial carcinoma.
