# Supplementary material for: ASPM, CDC20, DLGAP5, BUB1B, CDCA8, and NCAPG May Serve as Diagnostic and Prognostic Biomarkers in Endometrial Carcinoma
Source: Genet Res (Camb). 2022 Sep 17;2022:3217248. doi: 10.1155/2022/3217248 (PMC9509287; doi:10.1155/2022/3217248)
Supplement: Supplementary Materials — See Tables S1-S2 in the Supplementary Material. Table S1. Clinicopathological characteristics of UCEC patients with differential ASPM/CDC20/DLGAP5/BUB1B/CDCA8/NCAPG expression. UCEC, uterine corpus endometrial carcinoma. Table S2. Association of ASPM/CDC20/DLGAP5/BUB1B/CDCA8/NCAPG expression with clinicopathological characteristics in UCEC patients (single gene logistics regression analysis). UCEC, uterine corpus endometrial carcinoma. [file 3217248.f1.zip › 3217248.f1/Table S2 (1).docx]

| Characteristics | Total(N) | Odds Ratio(OR) | P value |
| --- | --- | --- | --- |
| Clinical stage (Stage III&Stage IV vs. Stage I&Stage II) | 552 | 1.401 (0.968-2.032) | 0.075 |
| Primary therapy outcome (PD&SD&PR vs. CR) | 480 | 1.725 (0.884-3.460) | 0.114 |
| Race (Asian&Black or African American vs. White) | 507 | 1.348 (0.902-2.021) | 0.146 |
| Age (>60 vs. <=60) | 549 | 1.557 (1.101-2.209) | 0.013 |
| Weight (>80 vs. <=80) | 528 | 0.640 (0.453-0.902) | 0.011 |
| Height (>160 vs. <=160) | 523 | 0.862 (0.611-1.215) | 0.396 |
| BMI (>30 vs. <=30) | 519 | 0.775 (0.545-1.100) | 0.154 |
| Histological type (Serous vs. Endometrioid) | 528 | 2.588 (1.693-4.011) | <0.001 |
| Residual tumor (R1&R2 vs. R0) | 413 | 1.443 (0.738-2.876) | 0.287 |
| Histologic grade (G3 vs. G1&G2) | 541 | 3.813 (2.655-5.520) | <0.001 |
| Tumor invasion(%) (>=50 vs. <50) | 474 | 1.284 (0.893-1.847) | 0.177 |
| Menopause status (Post vs. Pre&Peri) | 506 | 1.177 (0.662-2.106) | 0.579 |
| Hormones therapy (Yes vs. No) | 344 | 1.434 (0.774-2.699) | 0.255 |
| Diabetes (Yes vs. No) | 451 | 1.080 (0.713-1.637) | 0.715 |
| Radiation therapy (Yes vs. No) | 527 | 1.362 (0.967-1.921) | 0.078 |
| Surgical approach (Minimally Invasive vs. open) | 530 | 1.247 (0.880-1.769) | 0.216 |

**Table S2.1.** Association of ASPM expression with clinicopathological characteristics in UCEC patients (single gene logistics regression analysis). UCEC, uterine corpus endometrial carcinoma.

| Characteristics | Total(N) | Odds Ratio(OR) | P value |
| --- | --- | --- | --- |
| Clinical stage (Stage III&Stage IV vs. Stage I&Stage II) | 552 | 1.870 (1.288-2.732) | 0.001 |
| Primary therapy outcome (PD&SD&PR vs. CR) | 480 | 1.894 (0.967-3.846) | 0.068 |
| Race (Asian&Black or African American vs. White) | 507 | 1.152 (0.772-1.723) | 0.488 |
| Age (>60 vs. <=60) | 549 | 1.198 (0.848-1.695) | 0.306 |
| Weight (>80 vs. <=80) | 528 | 0.759 (0.538-1.069) | 0.115 |
| Height (>160 vs. <=160) | 523 | 0.839 (0.594-1.182) | 0.315 |
| BMI (>30 vs. <=30) | 519 | 0.794 (0.559-1.128) | 0.198 |
| Histological type (Serous vs. Endometrioid) | 528 | 4.360 (2.765-7.059) | <0.001 |
| Residual tumor (R1&R2 vs. R0) | 413 | 1.005 (0.513-1.969) | 0.988 |
| Histologic grade (G3 vs. G1&G2) | 541 | 5.963 (4.083-8.815) | <0.001 |
| Tumor invasion(%) (>=50 vs. <50) | 474 | 1.336 (0.930-1.923) | 0.117 |
| Menopause status (Post vs. Pre&Peri) | 506 | 1.413 (0.794-2.549) | 0.243 |
| Hormones therapy (Yes vs. No) | 344 | 0.688 (0.366-1.275) | 0.238 |
| Diabetes (Yes vs. No) | 451 | 0.857 (0.565-1.297) | 0.465 |
| Radiation therapy (Yes vs. No) | 527 | 1.715 (1.215-2.425) | 0.002 |
| Surgical approach (Minimally Invasive vs. open) | 530 | 1.224 (0.864-1.738) | 0.256 |

**Table S2.2.** Association of CDC20 expression with clinicopathological characteristics in UCEC patients (single gene logistics regression analysis). UCEC, uterine corpus endometrial carcinoma.

| Characteristics | Total(N) | Odds Ratio(OR) | P value |
| --- | --- | --- | --- |
| Clinical stage (Stage III&Stage IV vs. Stage I&Stage II) | 552 | 2.339 (1.603-3.441) | <0.001 |
| Primary therapy outcome (PD&SD&PR vs. CR) | 480 | 1.946 (0.993-3.953) | 0.057 |
| Race (Asian&Black or African American vs. White) | 507 | 1.348 (0.903-2.020) | 0.145 |
| Age (>60 vs. <=60) | 549 | 1.161 (0.822-1.642) | 0.396 |
| Weight (>80 vs. <=80) | 528 | 0.684 (0.484-0.964) | 0.030 |
| Height (>160 vs. <=160) | 523 | 0.741 (0.525-1.045) | 0.088 |
| BMI (>30 vs. <=30) | 519 | 0.938 (0.660-1.331) | 0.718 |
| Histological type (Serous vs. Endometrioid) | 528 | 3.538 (2.276-5.616) | <0.001 |
| Residual tumor (R1&R2 vs. R0) | 413 | 1.107 (0.565-2.167) | 0.766 |
| Histologic grade (G3 vs. G1&G2) | 541 | 5.739 (3.936-8.467) | <0.001 |
| Tumor invasion(%) (>=50 vs. <50) | 474 | 1.280 (0.891-1.842) | 0.182 |
| Menopause status (Post vs. Pre&Peri) | 506 | 1.306 (0.735-2.346) | 0.365 |
| Hormones therapy (Yes vs. No) | 344 | 0.746 (0.396-1.383) | 0.355 |
| Diabetes (Yes vs. No) | 451 | 1.046 (0.690-1.584) | 0.832 |
| Radiation therapy (Yes vs. No) | 527 | 1.588 (1.127-2.244) | 0.008 |
| Surgical approach (Minimally Invasive vs. open) | 530 | 1.338 (0.944-1.900) | 0.103 |

**Table S2.3.** Association of DLGAP5 expression with clinicopathological characteristics in UCEC patients (single gene logistics regression analysis). UCEC, uterine corpus endometrial carcinoma.

| Characteristics | Total(N) | Odds Ratio(OR) | P value |
| --- | --- | --- | --- |
| Clinical stage (Stage III&Stage IV vs. Stage I&Stage II) | 552 | 2.014 (1.385-2.947) | <0.001 |
| Primary therapy outcome (PD&SD&PR vs. CR) | 480 | 1.519 (0.781-3.016) | 0.222 |
| Race (Asian&Black or African American vs. White) | 507 | 1.363 (0.912-2.043) | 0.132 |
| Age (>60 vs. <=60) | 549 | 1.161 (0.822-1.642) | 0.396 |
| Weight (>80 vs. <=80) | 528 | 0.702 (0.497-0.989) | 0.043 |
| Height (>160 vs. <=160) | 523 | 0.836 (0.592-1.178) | 0.306 |
| BMI (>30 vs. <=30) | 519 | 0.826 (0.581-1.173) | 0.286 |
| Histological type (Serous vs. Endometrioid) | 528 | 2.563 (1.677-3.971) | <0.001 |
| Residual tumor (R1&R2 vs. R0) | 413 | 0.780 (0.391-1.523) | 0.470 |
| Histologic grade (G3 vs. G1&G2) | 541 | 3.587 (2.501-5.184) | <0.001 |
| Tumor invasion(%) (>=50 vs. <50) | 474 | 1.199 (0.834-1.724) | 0.327 |
| Menopause status (Post vs. Pre&Peri) | 506 | 1.295 (0.728-2.325) | 0.381 |
| Hormones therapy (Yes vs. No) | 344 | 0.835 (0.446-1.548) | 0.569 |
| Diabetes (Yes vs. No) | 451 | 0.976 (0.644-1.478) | 0.908 |
| Radiation therapy (Yes vs. No) | 527 | 1.242 (0.882-1.751) | 0.214 |
| Surgical approach (Minimally Invasive vs. open) | 530 | 1.240 (0.875-1.760) | 0.228 |

**Table S2.4.** Association of BUB1B expression with clinicopathological characteristics in UCEC patients (single gene logistics regression analysis). UCEC, uterine corpus endometrial carcinoma.

| Characteristics | Total(N) | Odds Ratio(OR) | P value |
| --- | --- | --- | --- |
| Clinical stage (Stage III&Stage IV vs. Stage I&Stage II) | 552 | 2.252 (1.545-3.309) | <0.001 |
| Primary therapy outcome (PD&SD&PR vs. CR) | 480 | 1.694 (0.869-3.397) | 0.127 |
| Race (Asian&Black or African American vs. White) | 507 | 1.201 (0.804-1.797) | 0.371 |
| Age (>60 vs. <=60) | 549 | 1.374 (0.972-1.945) | 0.073 |
| Weight (>80 vs. <=80) | 528 | 0.629 (0.445-0.887) | 0.008 |
| Height (>160 vs. <=160) | 523 | 0.849 (0.602-1.197) | 0.352 |
| BMI (>30 vs. <=30) | 519 | 0.704 (0.494-1.000) | 0.050 |
| Histological type (Serous vs. Endometrioid) | 528 | 3.573 (2.298-5.672) | <0.001 |
| Residual tumor (R1&R2 vs. R0) | 413 | 1.269 (0.650-2.510) | 0.487 |
| Histologic grade (G3 vs. G1&G2) | 541 | 5.057 (3.486-7.418) | <0.001 |
| Tumor invasion(%) (>=50 vs. <50) | 474 | 1.332 (0.927-1.917) | 0.121 |
| Menopause status (Post vs. Pre&Peri) | 506 | 1.080 (0.607-1.927) | 0.793 |
| Hormones therapy (Yes vs. No) | 344 | 0.750 (0.401-1.389) | 0.362 |
| Diabetes (Yes vs. No) | 451 | 1.008 (0.666-1.527) | 0.969 |
| Radiation therapy (Yes vs. No) | 527 | 1.518 (1.077-2.144) | 0.017 |
| Surgical approach (Minimally Invasive vs. open) | 530 | 1.164 (0.821-1.651) | 0.395 |

**Table S2.5.** Association of CDCA8 expression with clinicopathological characteristics in UCEC patients (single gene logistics regression analysis). UCEC, uterine corpus endometrial carcinoma.

| Characteristics | Total(N) | Odds Ratio(OR) | P value |
| --- | --- | --- | --- |
| Clinical stage (Stage III&Stage IV vs. Stage I&Stage II) | 552 | 1.560 (1.077-2.268) | 0.019 |
| Primary therapy outcome (PD&SD&PR vs. CR) | 480 | 1.402 (0.722-2.762) | 0.320 |
| Race (Asian&Black or African American vs. White) | 507 | 1.581 (1.057-2.377) | 0.027 |
| Age (>60 vs. <=60) | 549 | 1.148 (0.812-1.623) | 0.434 |
| Weight (>80 vs. <=80) | 528 | 0.736 (0.521-1.037) | 0.080 |
| Height (>160 vs. <=160) | 523 | 0.788 (0.559-1.112) | 0.176 |
| BMI (>30 vs. <=30) | 519 | 0.810 (0.570-1.149) | 0.238 |
| Histological type (Serous vs. Endometrioid) | 528 | 2.006 (1.322-3.072) | 0.001 |
| Residual tumor (R1&R2 vs. R0) | 413 | 1.282 (0.657-2.537) | 0.467 |
| Histologic grade (G3 vs. G1&G2) | 541 | 5.147 (3.549-7.546) | <0.001 |
| Tumor invasion(%) (>=50 vs. <50) | 474 | 1.221 (0.850-1.756) | 0.280 |
| Menopause status (Post vs. Pre&Peri) | 506 | 1.099 (0.618-1.961) | 0.747 |
| Hormones therapy (Yes vs. No) | 344 | 0.656 (0.347-1.219) | 0.187 |
| Diabetes (Yes vs. No) | 451 | 0.933 (0.616-1.413) | 0.744 |
| Radiation therapy (Yes vs. No) | 527 | 1.488 (1.056-2.102) | 0.023 |
| Surgical approach (Minimally Invasive vs. open) | 530 | 1.092 (0.771-1.549) | 0.620 |

**Table S2.6.** Association of NCAPG expression with clinicopathological characteristics in UCEC patients (single gene logistics regression analysis). UCEC, uterine corpus endometrial carcinoma.
